# Supplementary material for: The growth factor EPIREGULIN promotes basal progenitor cell proliferation in the developing neocortex
Source: EMBO J. 2024 Mar 21;43(8):2. doi: 10.1038/s44318-024-00068-7 (PMC11021537; doi:10.1038/s44318-024-00068-7)
Supplement: Supplementary file 4 — Appendix [file 44318_2024_68_MOESM4_ESM.pdf]

The growth factor EPIREGULIN promotes basal progenitor cell proliferation in the developing neocortex

Paula Cubillos, Nora Ditzer, Annika Kolodziejczyk, Gustav Schwenk, Janine Hoffmann, Theresa M. Schütze, Razvan P. Derihaci, Cahit Birdir, Johannes E. M. Köllner, Andreas Petzold, Mihail Sarov, Ulrich Martin, Katherine R. Long, Pauline Wimberger, Mareike Albert

APPENDIX TABLE OF CONTENTS

Appendix Figure S1. Analysis of Epiregulin protein expression (page 2).

Appendix Figure S2. Expression, DNA methylation and genomics of *EREG* in brain tumours (page 3).

Appendix Figure S3. Comparison of EPIREGULIN treatment using mNcx slice and HERO cultures (page 4).

Appendix Figure S4. Epidermal growth factor and receptor expression (page 5).

Appendix Figure S5. Treatment of human cortical organoids with receptor inhibitors (page 6).

Appendix Figure S6. Conservation of putative *EREG* CREs in different species (page 7).

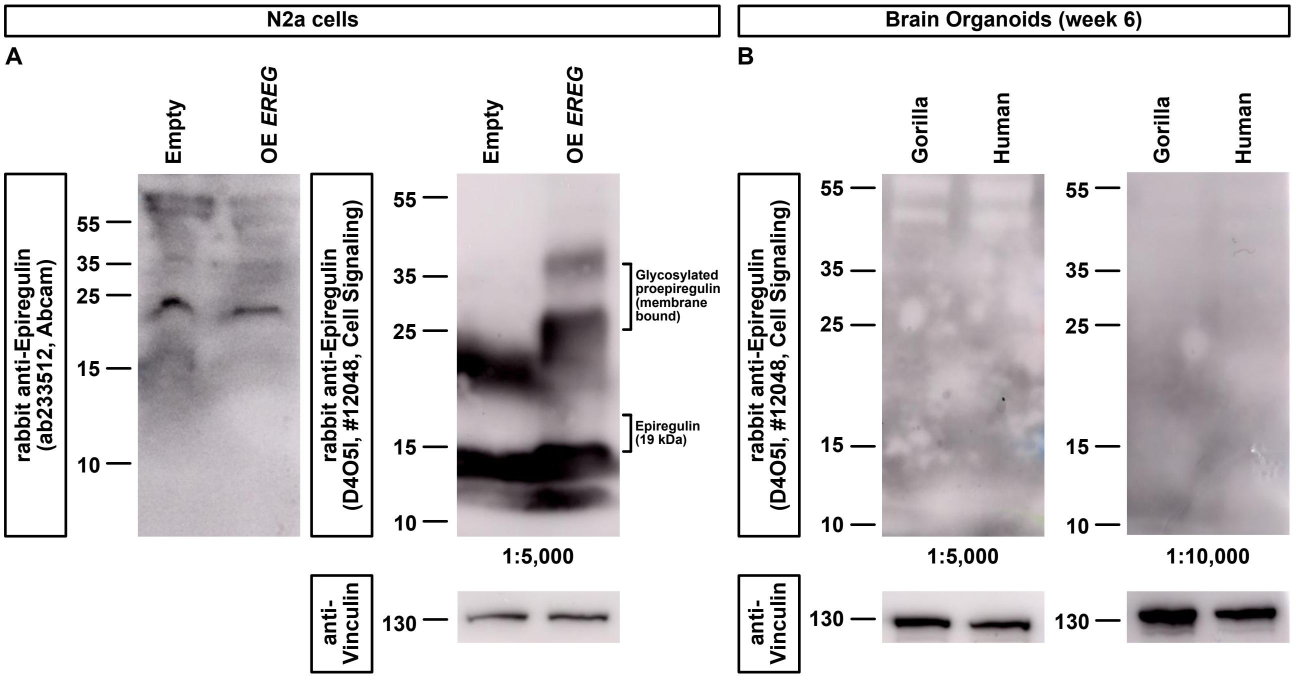

Appendix Figure S1. Analysis of EPIREGULIN protein expression.

A EPIREGULIN protein expression in mouse N2a cells transfected with an empty plasmid (control) or a plasmid over-expressing human EPIREGULIN, analyzed by Western blotting. Protein lysates were analyzed 24 hours after trans-fection. Two different antibodies directed against human EPIREGULIN were tested. Vinculin was used as loading control. The sizes of marker proteins are indicated on the left of each blot (in kDa).

B EPIREGULIN protein expression in gorilla cerebral and human cortical organoids analyzed by Western blotting. Two different antibody concentrations are shown. Vinculin was used as loading control.

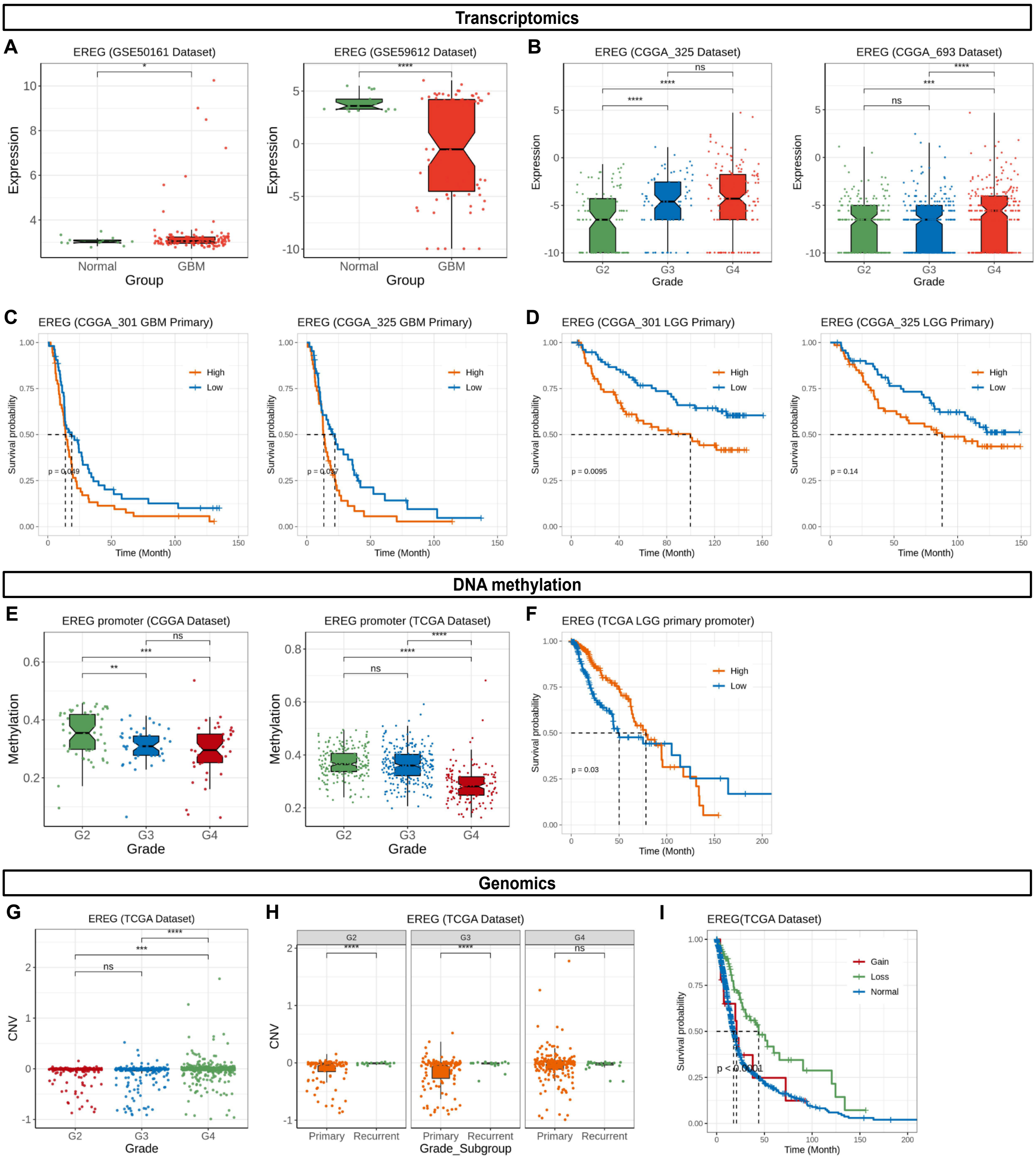

Appendix Figure S2. Expression, DNA methylation and genomics of EREG in brain tumors.

A Expression of EREG in normal versus glioblastoma (GBM) tissue based on microarray and RNA-seq data (Gill et al, 2014; Griesinger et al, 2013).

B Expression of EREG in grade 2 glioma (G2), G3 and G4 based on RNA-seq data (Bao et al, 2014).

C, D Survival probability in relation to low (blue) and high (orange) EREG expression detected by microarray (Sun et al, 2014; Yan et al, 2012) or RNA-seq (Bao et al., 2014) upon glioblastoma multiforme (C; GBM) and primary low-grade glioma (D; LGG).

E Promoter DNA methylation of EREG in G2, G3 and G4 glioma based on DNA methylation profiling (Ceccarelli et al, 2016; Zhang et al, 2013).

F Survival probability in relation to low (blue) and high (orange) EREG promoter DNA methylation primary low-grade glioma (Ceccarelli et al., 2016).

G, H Copy number variation (CNV) associated with the EREG locus in G2, G3 and G4 glioma (G) and primary versus recurrent glioma (H) (Ceccarelli et al., 2016).

I Survival probability in relation to gain (red), normal (blue) and loss (green) of EREG (Ceccarelli et al., 2016). All data was extracted from BrainBase (https://ngdc.cncb.ac.cn/brainbase/).

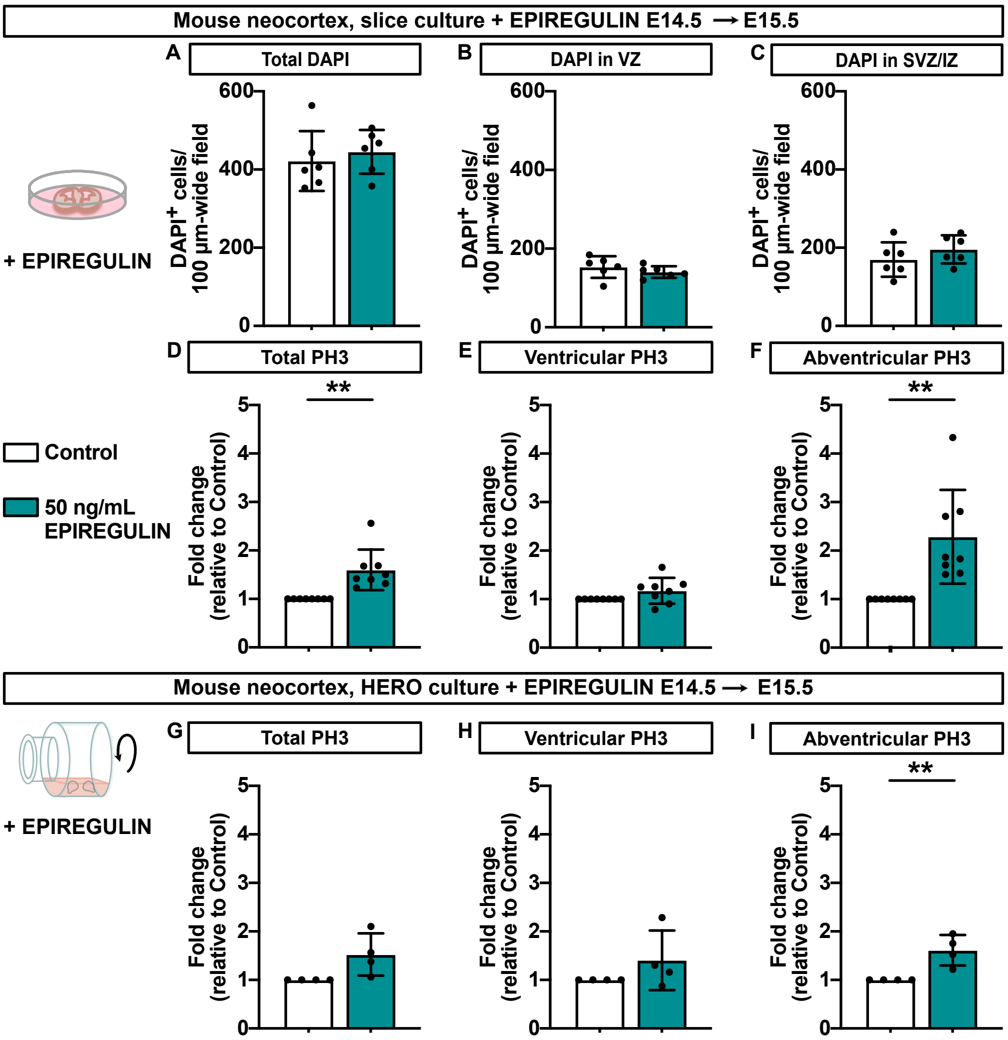

Appendix Figure S3. Comparison of EPIREGULIN treatment using mNcx slice and HERO cultures.

A–C Quantifications of total DAPI+ cells, DAPI positive cells in the VZ and DAPI positive cells in the SVZ/IZ of mNcx slice cultures treated with 50 ng/mL of EPIREGULIN.

D–F Quantifications of total, ventricular and abventricular mitotic PH3+ cells of mNcx slice cultures treated with 50 ng/mL of EPIREGULIN.

G–H Quantifications of total, ventricular and abventricular mitotic PH3+ cells of mNcx hemispheres in HERO cultures treated with 50 ng/mL of EPIREGULIN.

Data information: Bar graphs represent mean values. Error bars represent SD; A–C, of 8 embryos from different litters, with each dot representing the average of 3–6 images of different sections of the same brain; D–F, of 4 embryos from different litters with each dot representing the average of 3 images of different sections of the same brain. ** $p < 0.01$; Student's t-test.

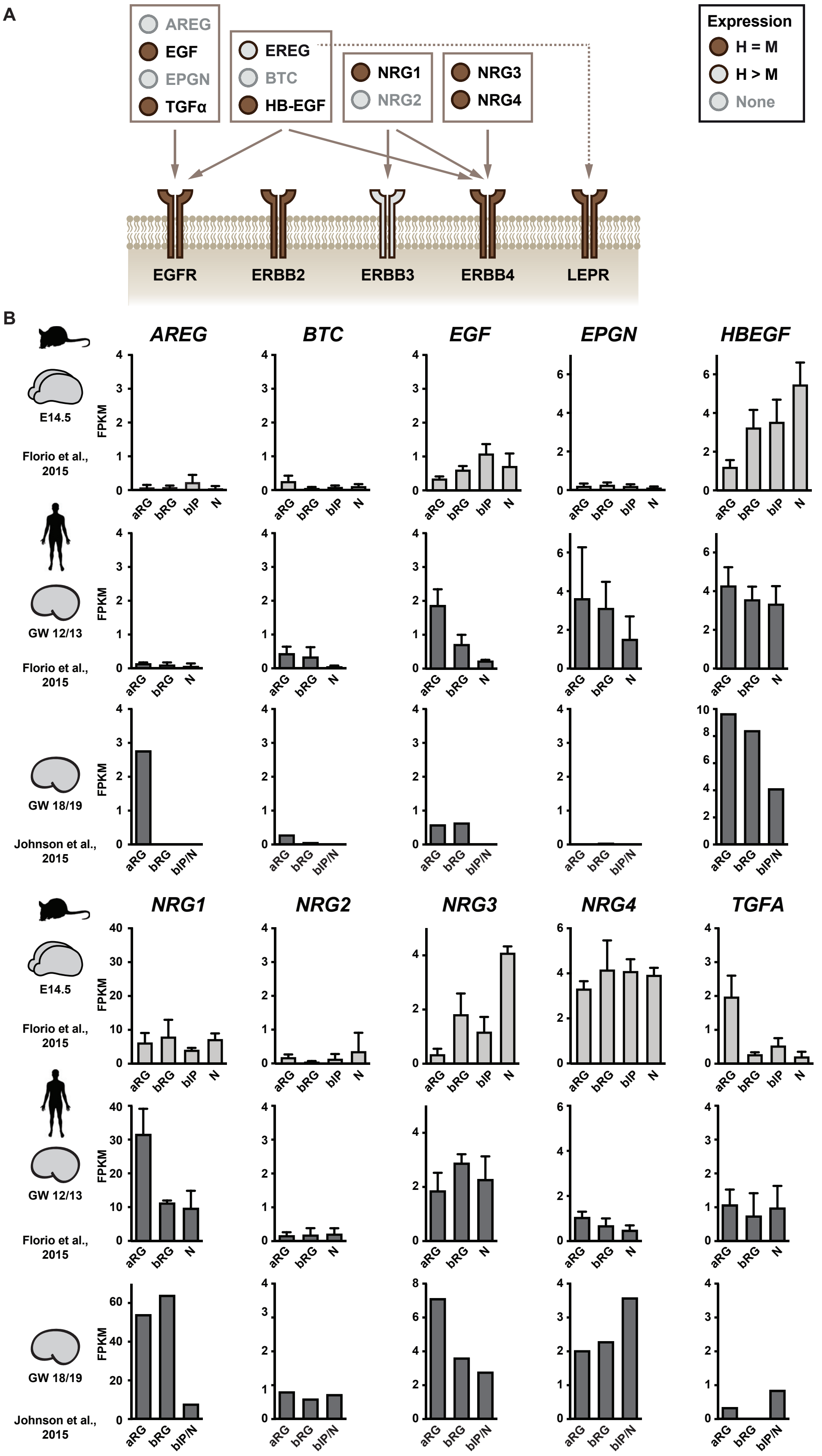

Appendix Figure S4. Epidermal growth factor and receptor expression.

A Schematic illustration of the epidermal growth factors and their respective receptors. Colors indicated mRNA expression: brown, similar in mouse and human; grey with dark contour, higher expression in human RG than in mouse; grey, no expression in both species.

B Expression of epidermal growth factor genes in mouse (top) and human (bottom) NPCs and neurons analyzed by RNA-seq (data from Florio et al. (2015); Johnson et al. (2015)) at the indicated developmental stages.

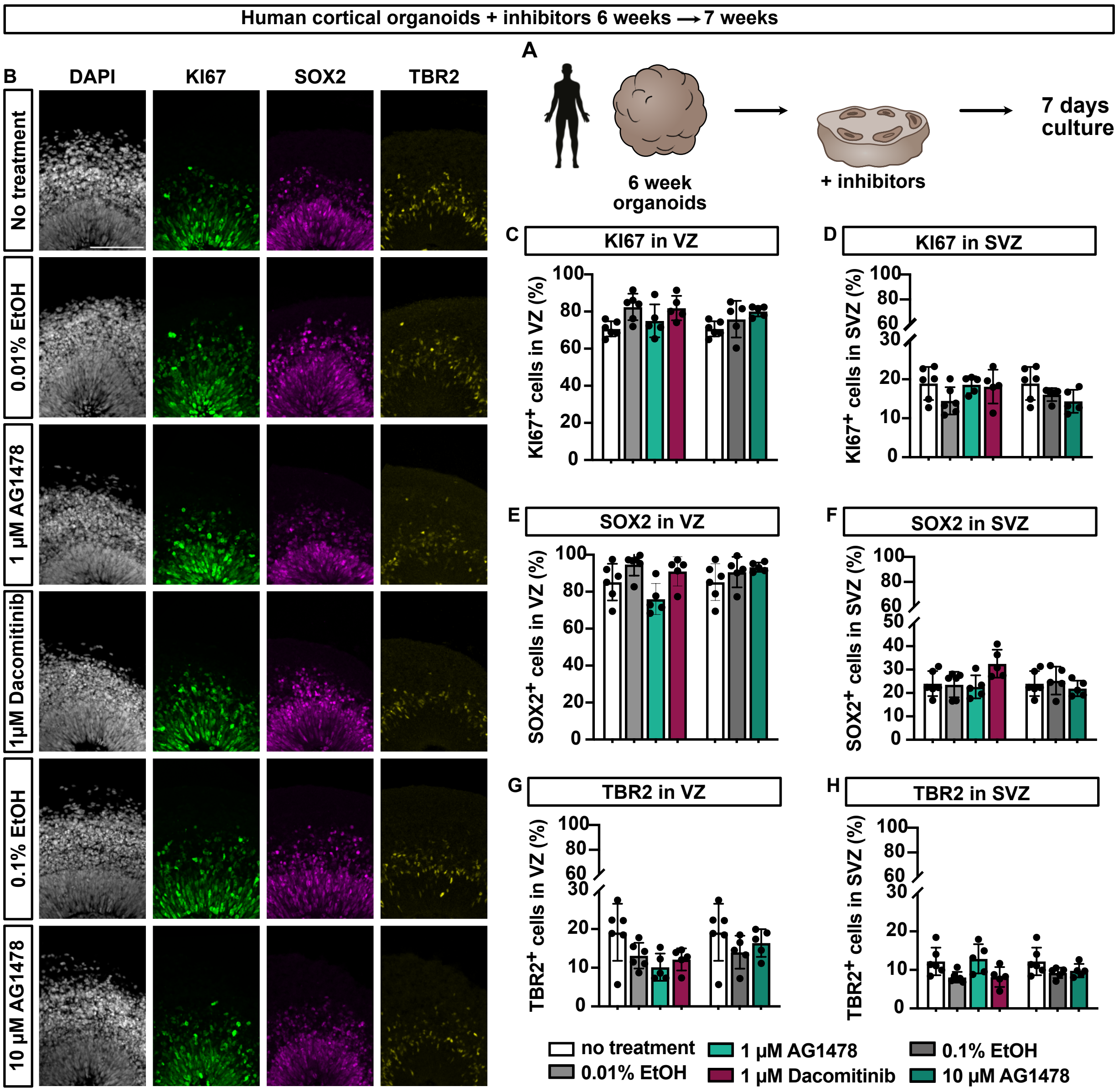

Appendix Figure S5. Treatment of human cortical organoids with receptor inhibitors.

A Schematic of experimental workflow. Human cortical organoids (6 week) were cultured in the presence of EGFR and Erbb4 receptor inhibitors for 7 days.

B DAPI staining and immunofluorescence for KI67, SOX2 and TBR2.

C–H Quantifications of KI67, SOX2 and TBR2 in the VZ and SVZ of human cortical organoids.

Data information: Scale bar, 100 µm. Bar graphs represent mean values. Error bars represent SD of 5–6 cortical organoids; one-way ANOVA with Dunnett post hoc test; no statistically significant changes were detected.

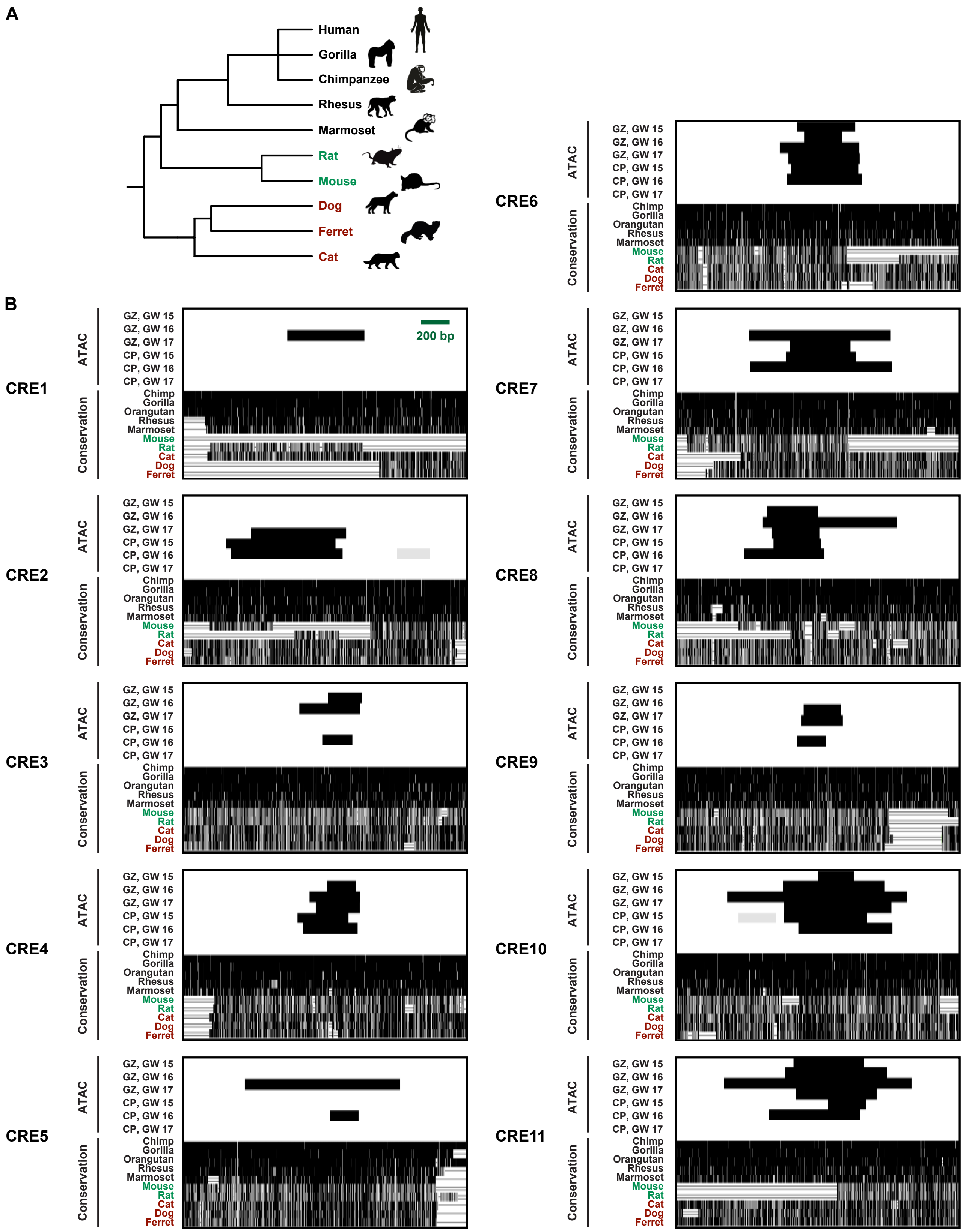

Appendix Figure S6. Conservation of putative EREG CREs in different species.
A Phylogenetic relationship between primates, rodents and carnivores, generated using phyloT.
B Evolutionary conservation of putative EREG CREs from 100 vertebrate species (Blanchette et al., 2004) for the indicated species. ATAC-seq (de la Torre-Ubieta et al., 2018) peaks are shown for reference.
