# Supplementary figures and images for: The growth factor EPIREGULIN promotes basal progenitor cell proliferation in the developing neocortex
Source: EMBO J. 2024 Mar 21;43(8):2. doi: 10.1038/s44318-024-00068-7 (PMC11021537; doi:10.1038/s44318-024-00068-7)

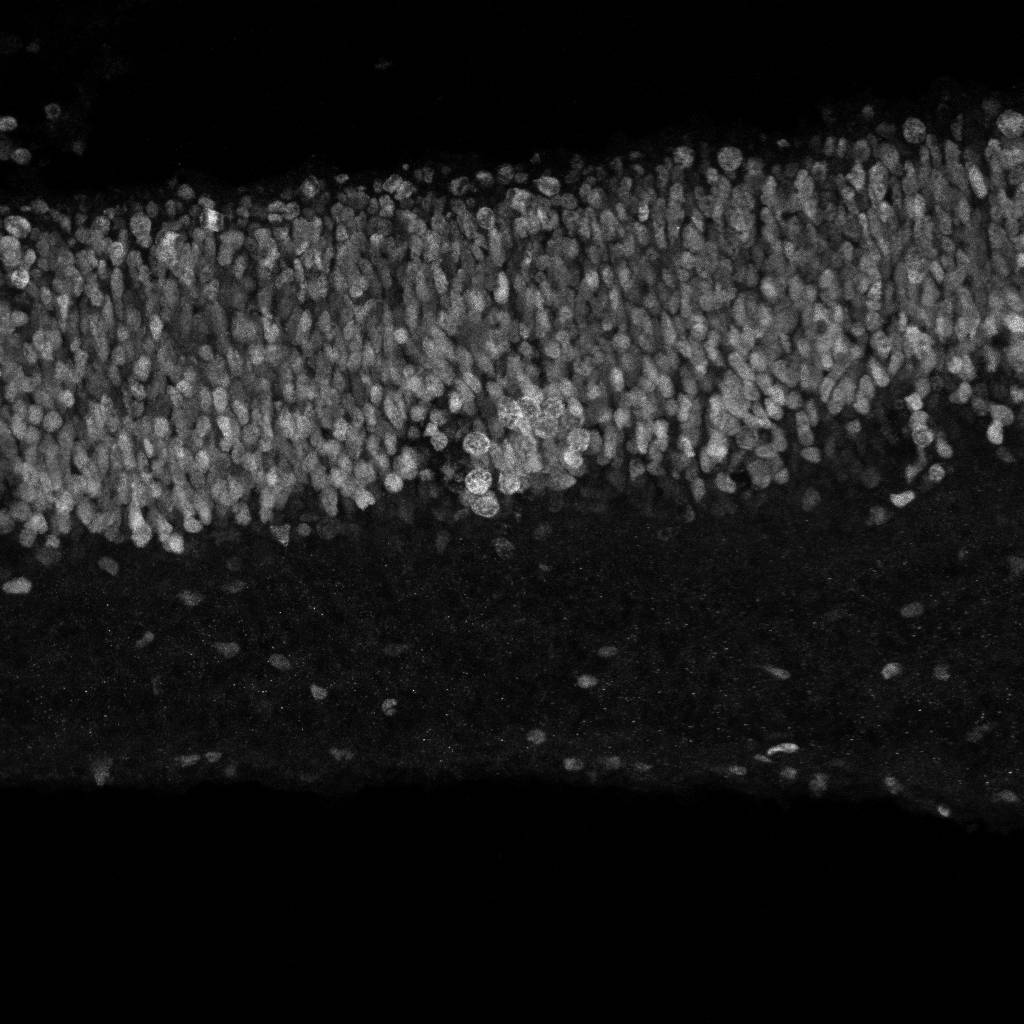

Supplement: Supplementary file 5 — Source Data Fig. 1 [file 44318_2024_68_MOESM5_ESM.zip › Figure 1/1F/Fig1F_50ng_DAPI,PH3(488),SOX2(555).tif]

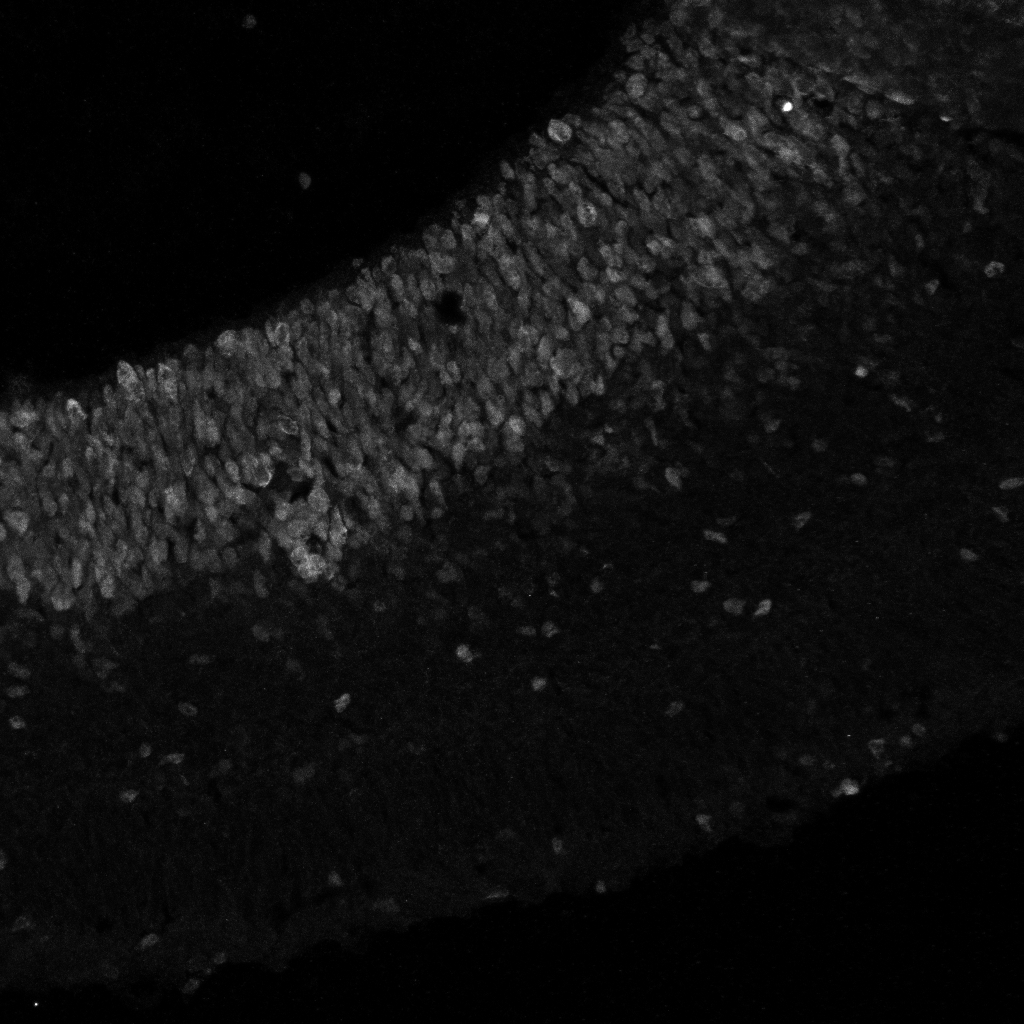

Supplement: Supplementary file 5 — Source Data Fig. 1 [file 44318_2024_68_MOESM5_ESM.zip › Figure 1/1F/Fig1F_ctrl_DAPI,PH3(488),SOX2(555).tif]

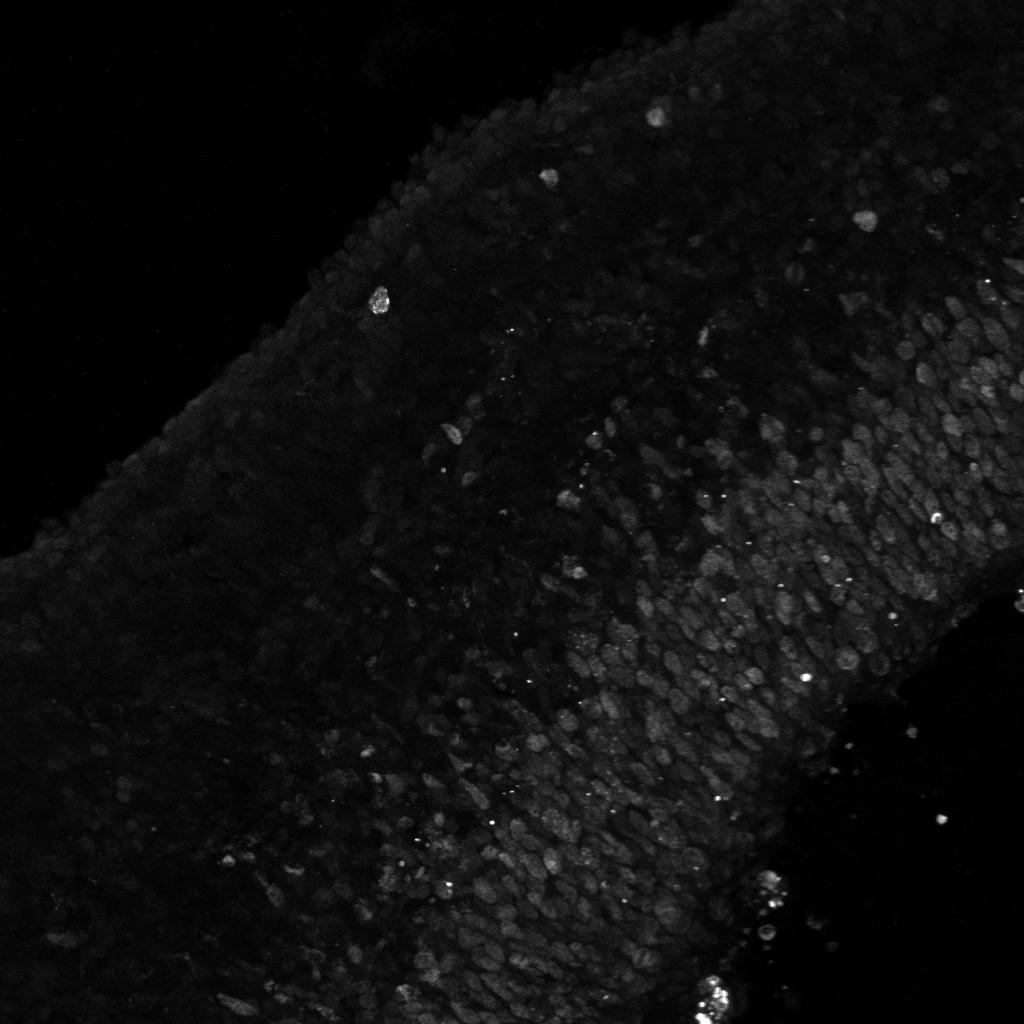

Supplement: Supplementary file 5 — Source Data Fig. 1 [file 44318_2024_68_MOESM5_ESM.zip › Figure 1/1M/Fig1M_50ng_DAPI,Ki67(488),PCNA(647).tif]

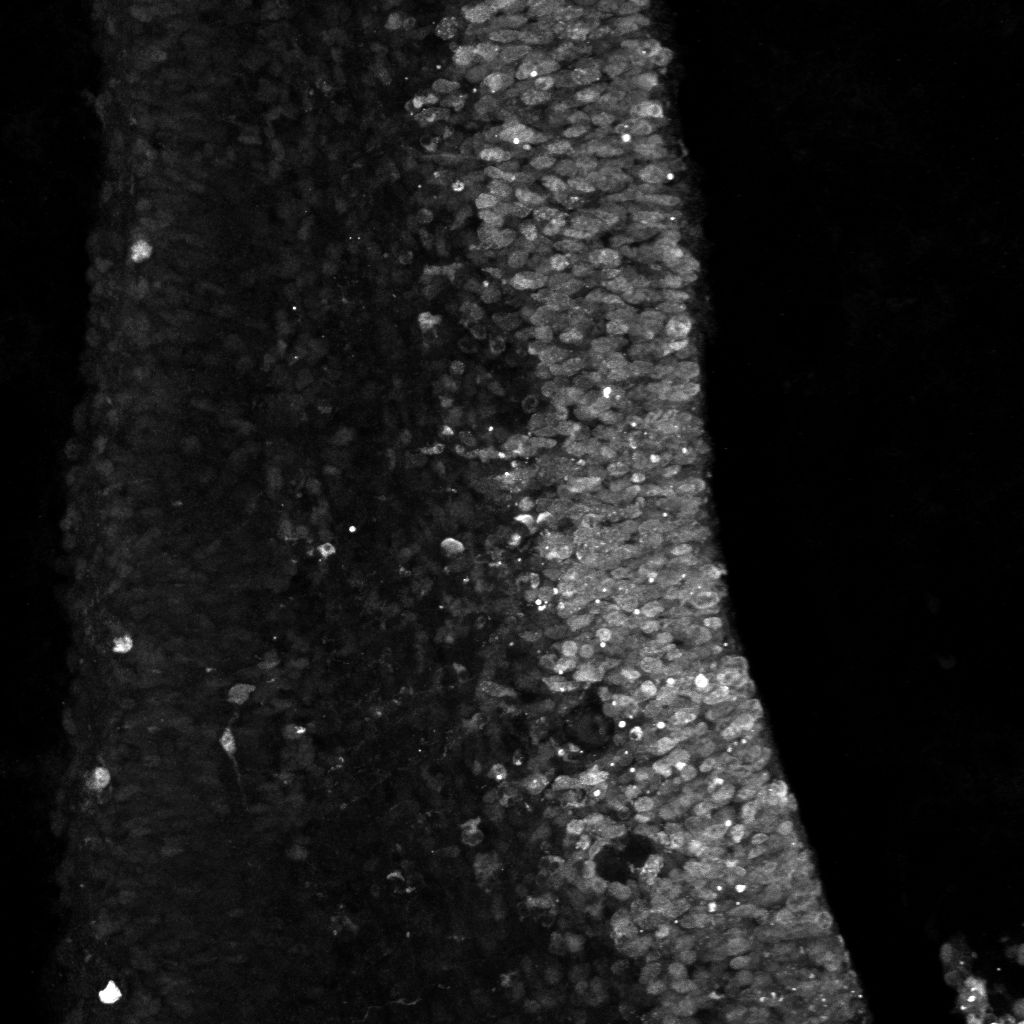

Supplement: Supplementary file 5 — Source Data Fig. 1 [file 44318_2024_68_MOESM5_ESM.zip › Figure 1/1M/Fig1M_ctrl_DAPI,Ki67(488),PCNA(647).tif]

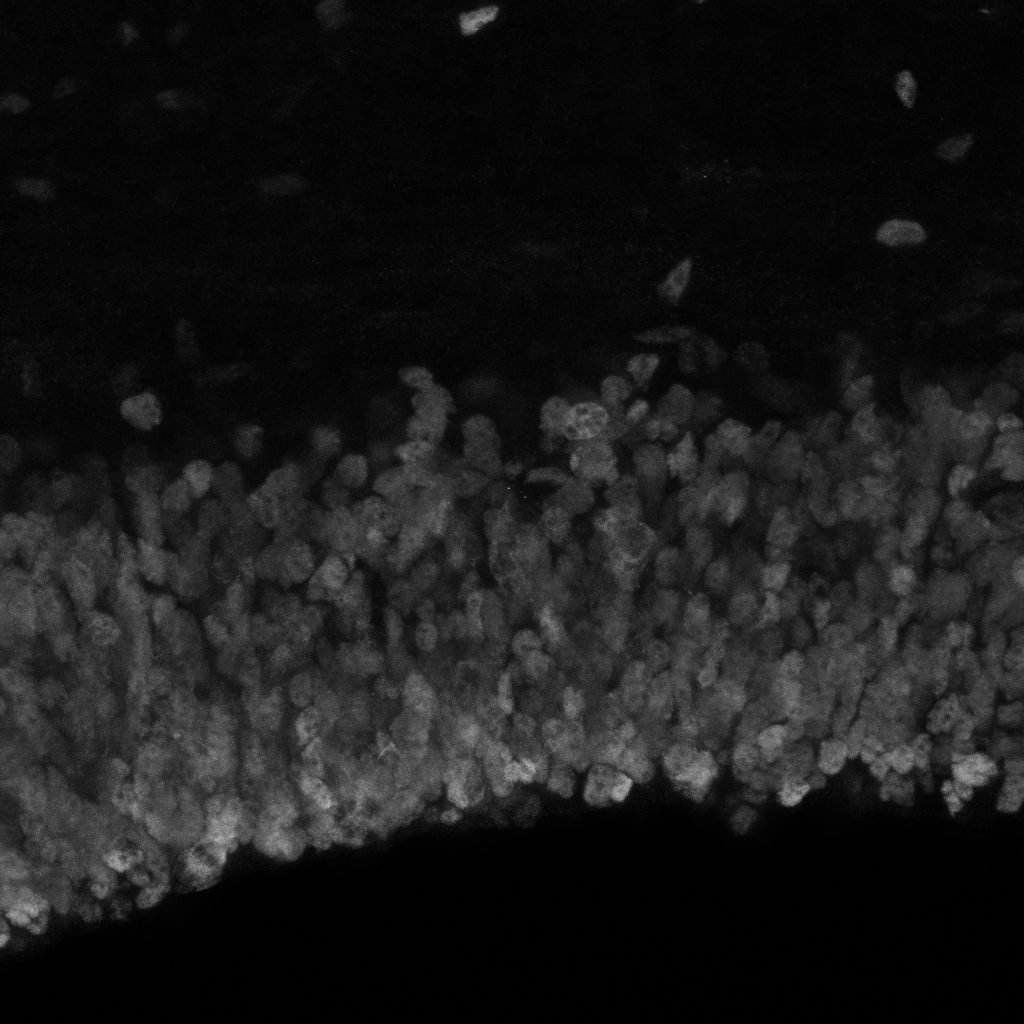

Supplement: Supplementary file 6 — Source Data Fig. 2 [file 44318_2024_68_MOESM6_ESM.zip › Figure 2/2I/Fig 2I_40x_DAPI,Pvim(488),Sox2(555).tif]

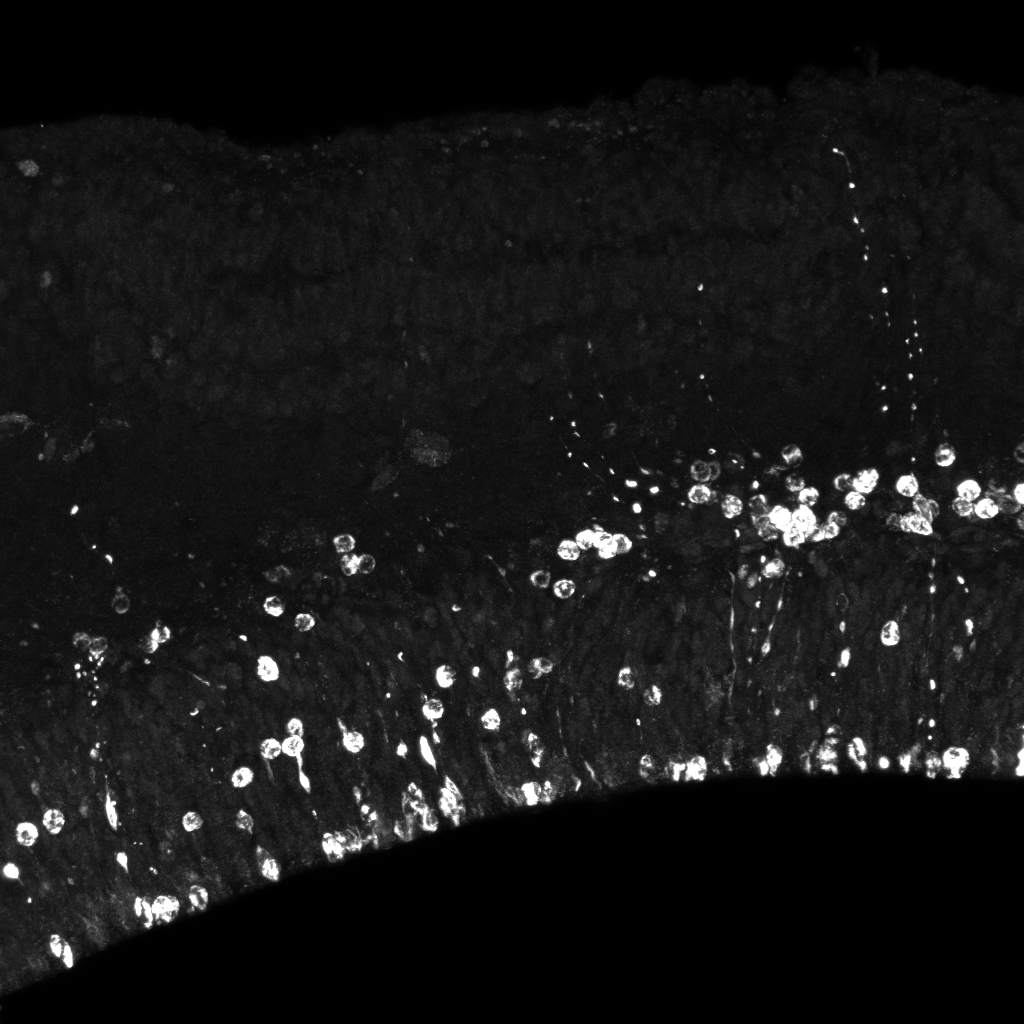

Supplement: Supplementary file 6 — Source Data Fig. 2 [file 44318_2024_68_MOESM6_ESM.zip › Figure 2/2I/Fig 2I_50ng_DAPI,Sox2(555),Pvim(647).tif]

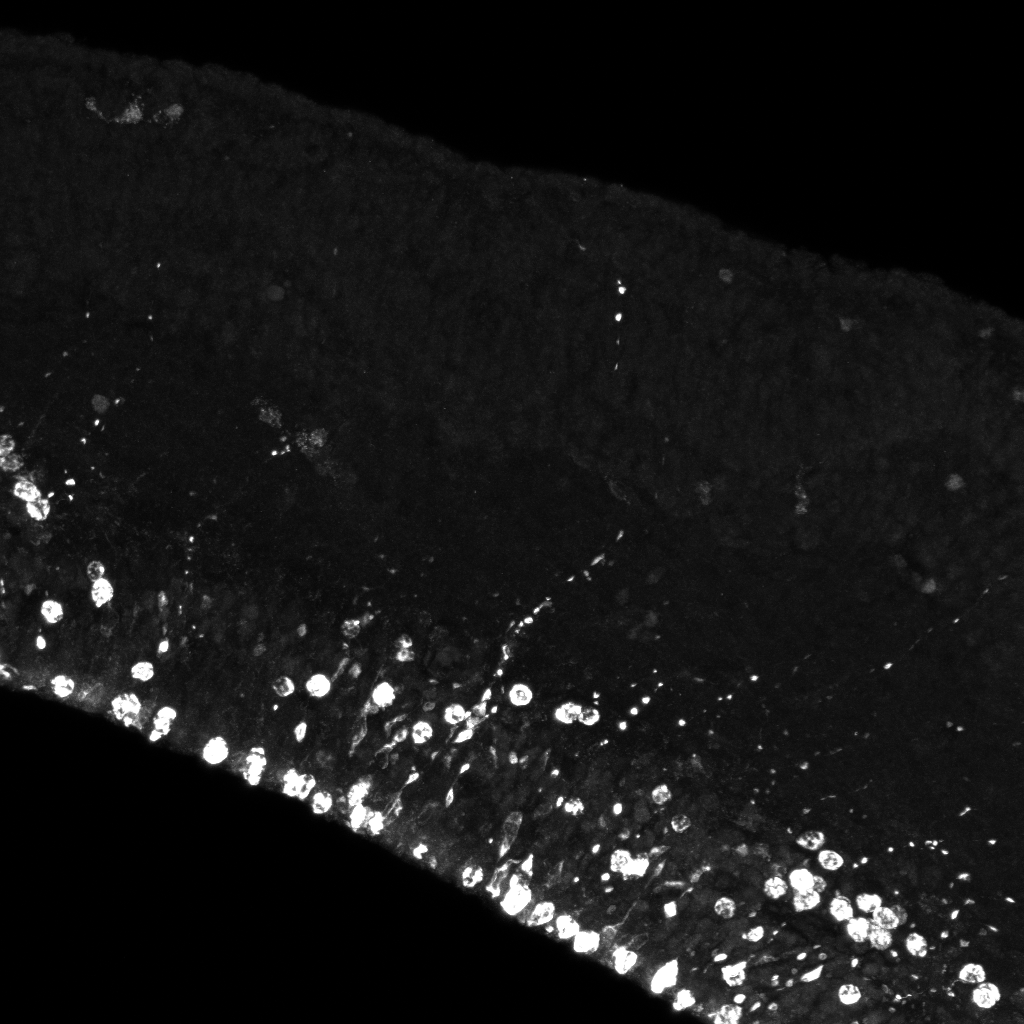

Supplement: Supplementary file 6 — Source Data Fig. 2 [file 44318_2024_68_MOESM6_ESM.zip › Figure 2/2I/Fig 2I_ctrl_DAPI,Sox2(555),Pvim(647.tif]

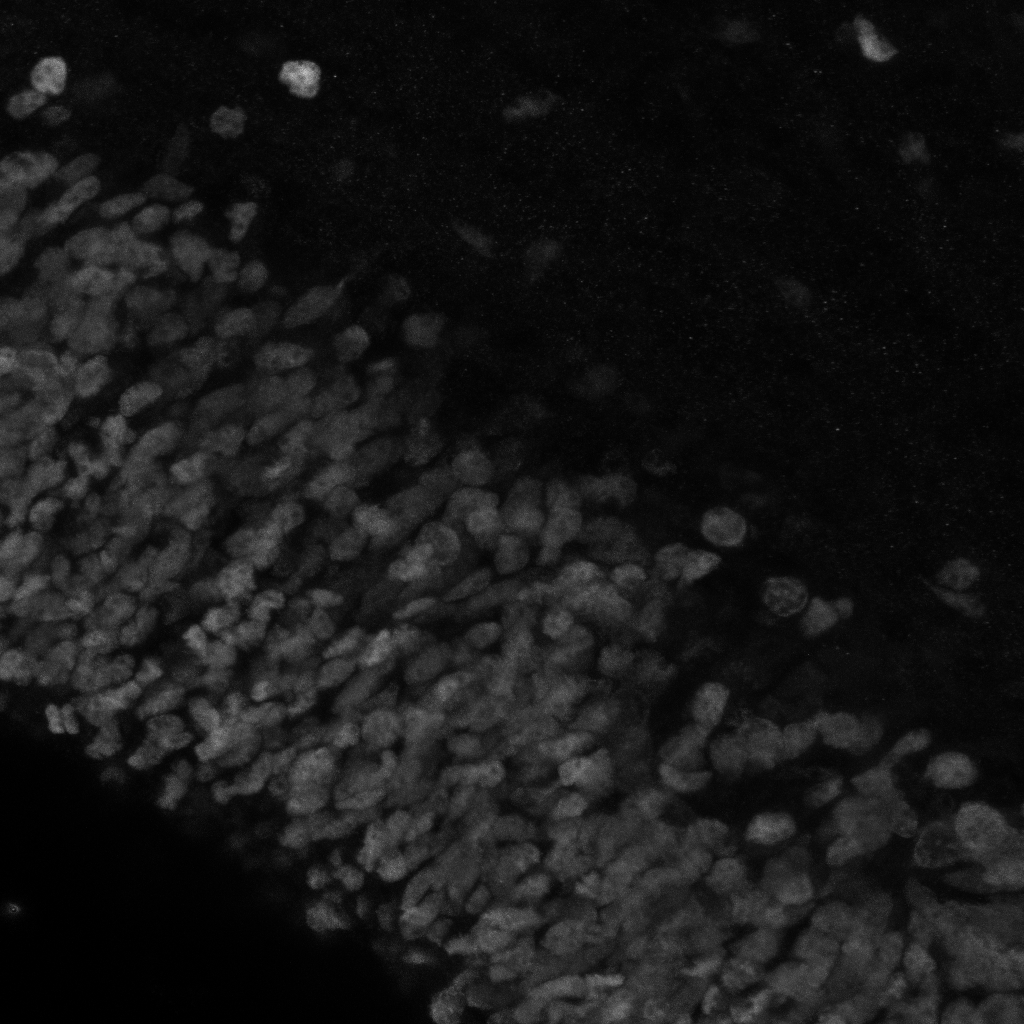

Supplement: Supplementary file 6 — Source Data Fig. 2 [file 44318_2024_68_MOESM6_ESM.zip › Figure 2/2I/Fig 2I_40x2_DAPI,Pvim(488),Sox2(555).tif]

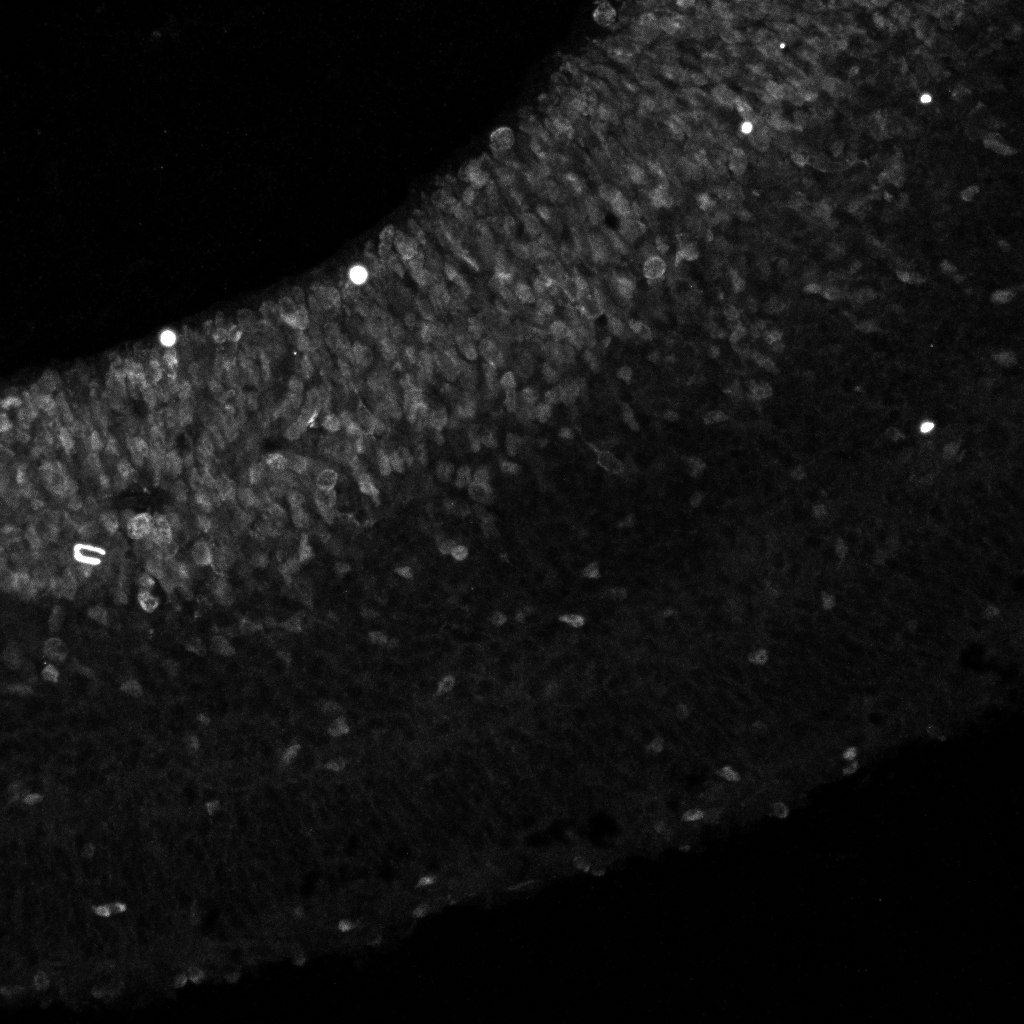

Supplement: Supplementary file 6 — Source Data Fig. 2 [file 44318_2024_68_MOESM6_ESM.zip › Figure 2/2A/Fig 2A_ctrl_DAPI,Tbr2(488),Sox2(555).tif]

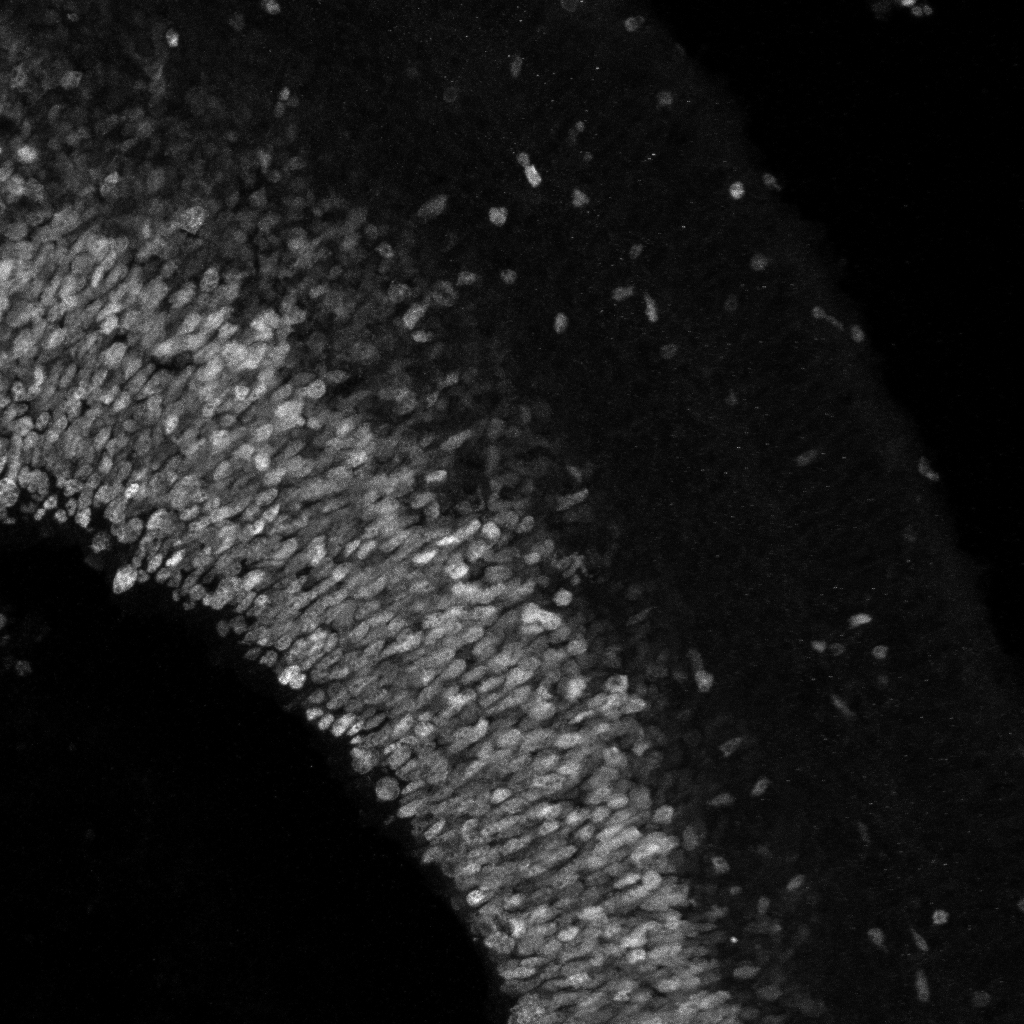

Supplement: Supplementary file 6 — Source Data Fig. 2 [file 44318_2024_68_MOESM6_ESM.zip › Figure 2/2A/Fig 2A_50ng_DAPI,Tbr2(488),Sox2(555).tif]

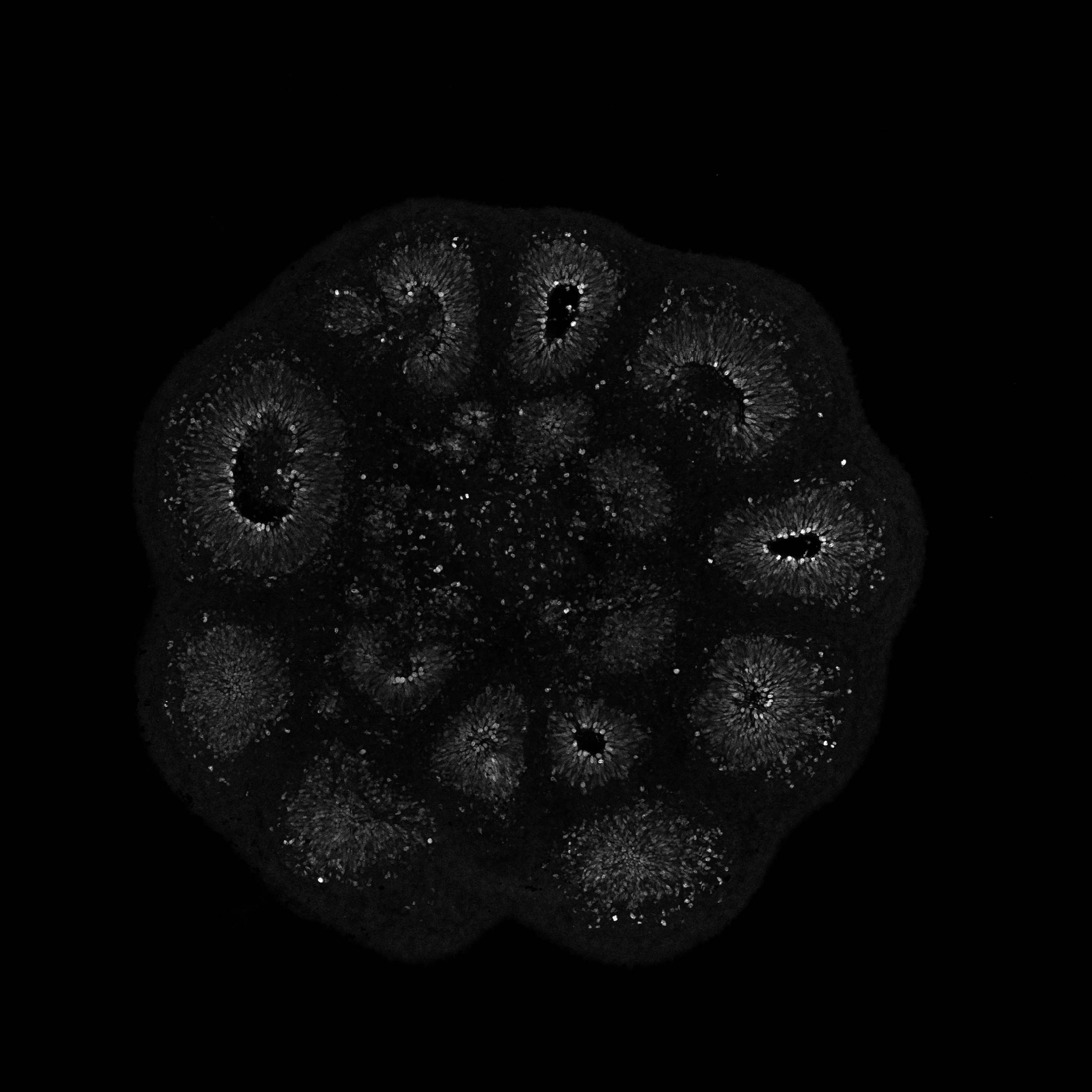

Supplement: Supplementary file 7 — Source Data Fig. 3 [file 44318_2024_68_MOESM7_ESM.zip › Figure 3/3B/Fig3B_whole org_DAPI, GFP(488), SOX2(555), KI67(647).tif]

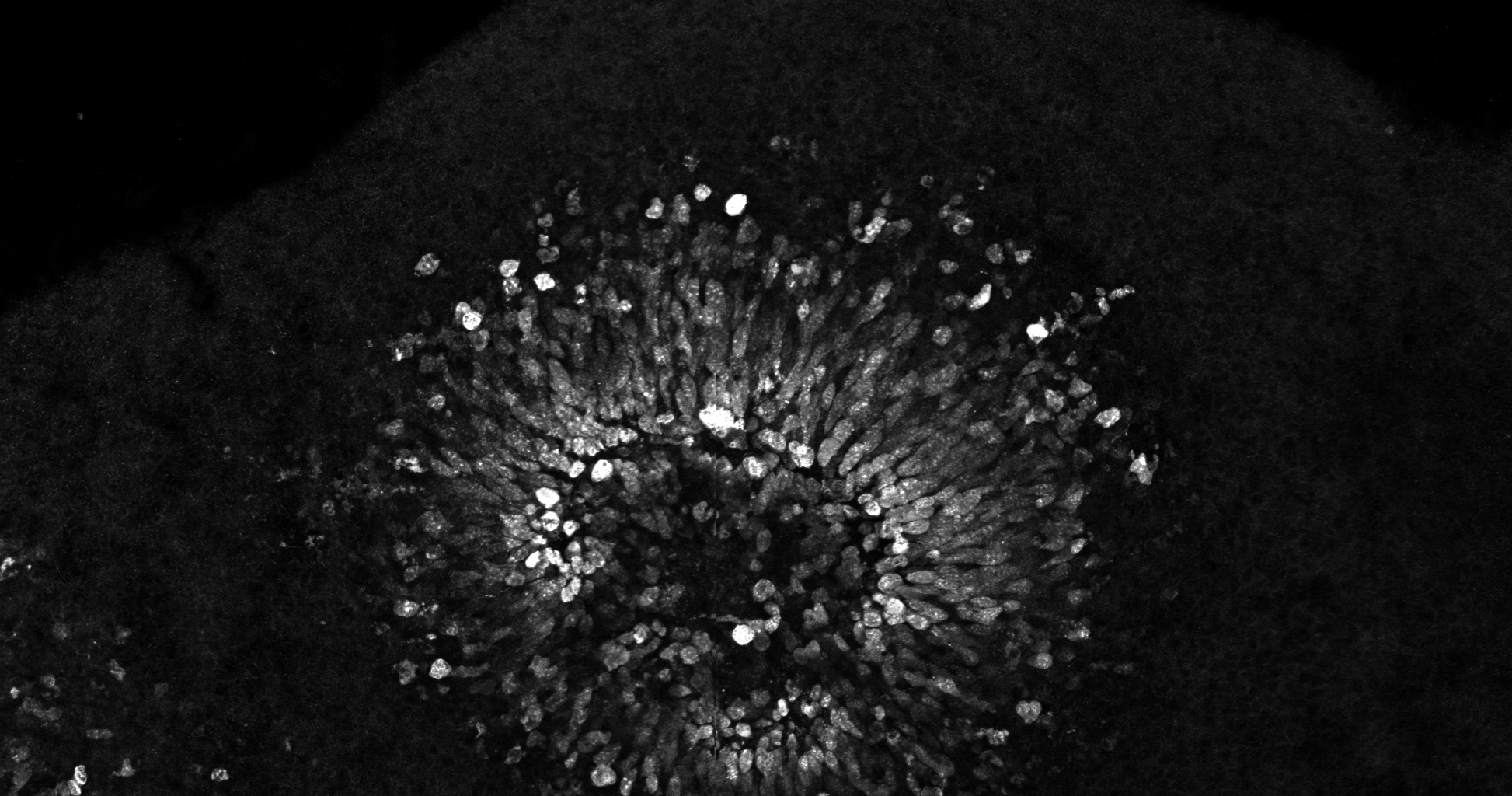

Supplement: Supplementary file 7 — Source Data Fig. 3 [file 44318_2024_68_MOESM7_ESM.zip › Figure 3/3C/Fig3C_gEREG KO1+2_DAPI, GFP(488),SOX2(555), KI67(647).tif]

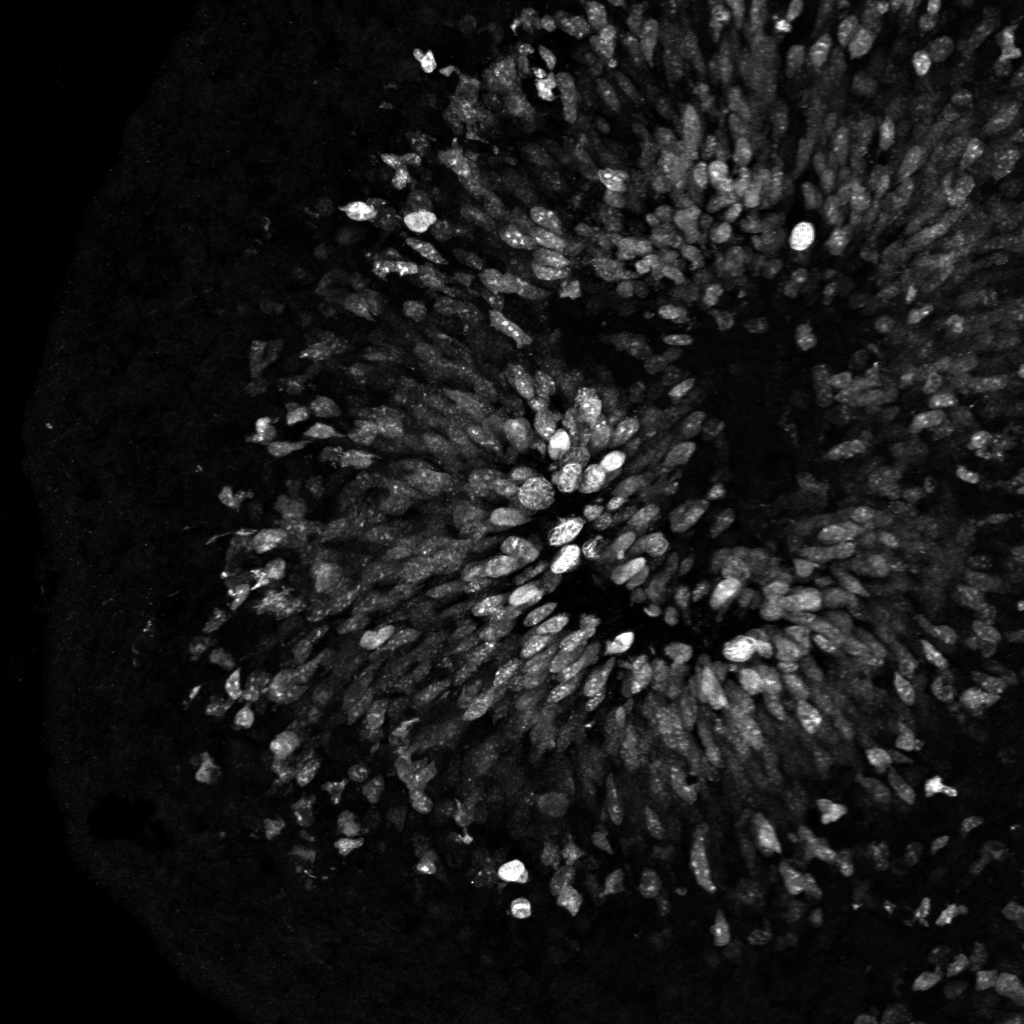

Supplement: Supplementary file 7 — Source Data Fig. 3 [file 44318_2024_68_MOESM7_ESM.zip › Figure 3/3C/Fig3C_gLacZ_DAPI, GFP(488),SOX2(555), KI67(647).tif]

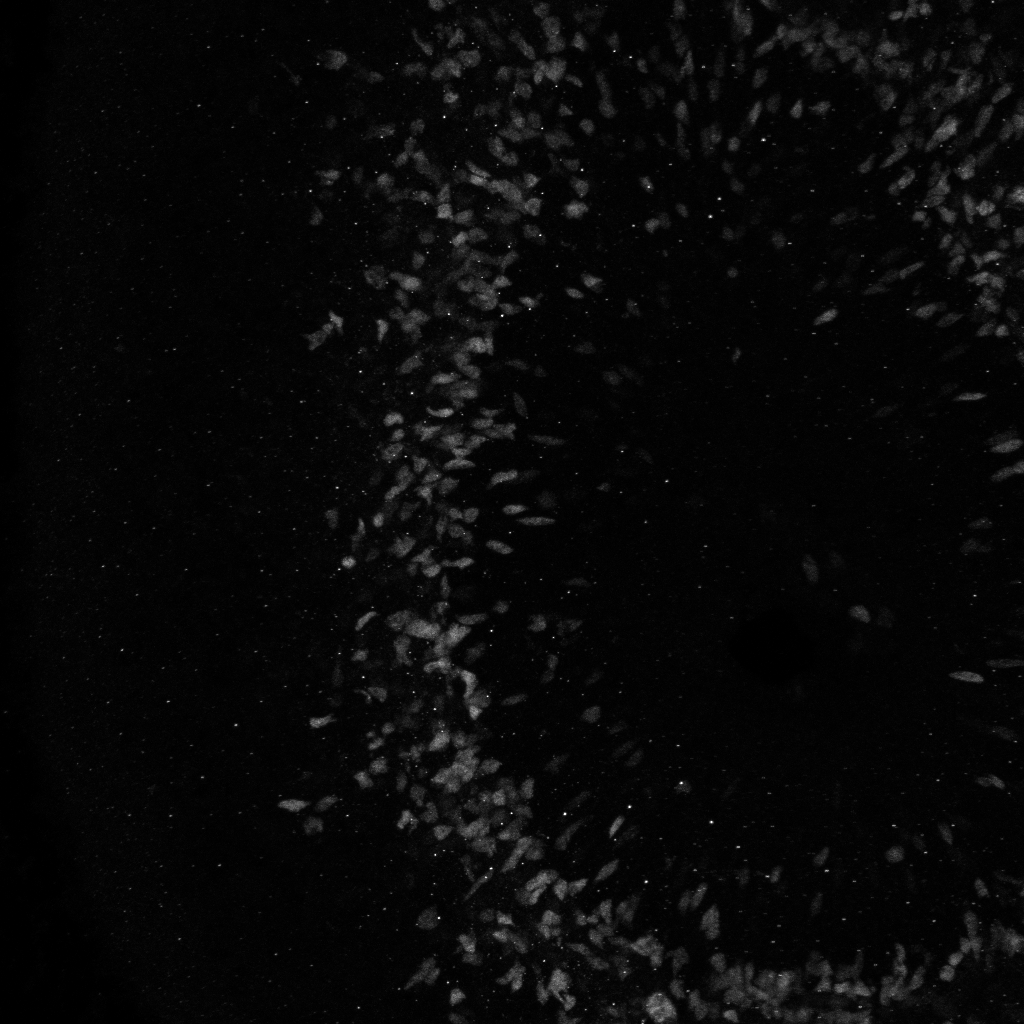

Supplement: Supplementary file 7 — Source Data Fig. 3 [file 44318_2024_68_MOESM7_ESM.zip › Figure 3/3F/Fig3F_gLacZ_DAPI, GFP(488),SOX2(555), TBR2(647).tif]

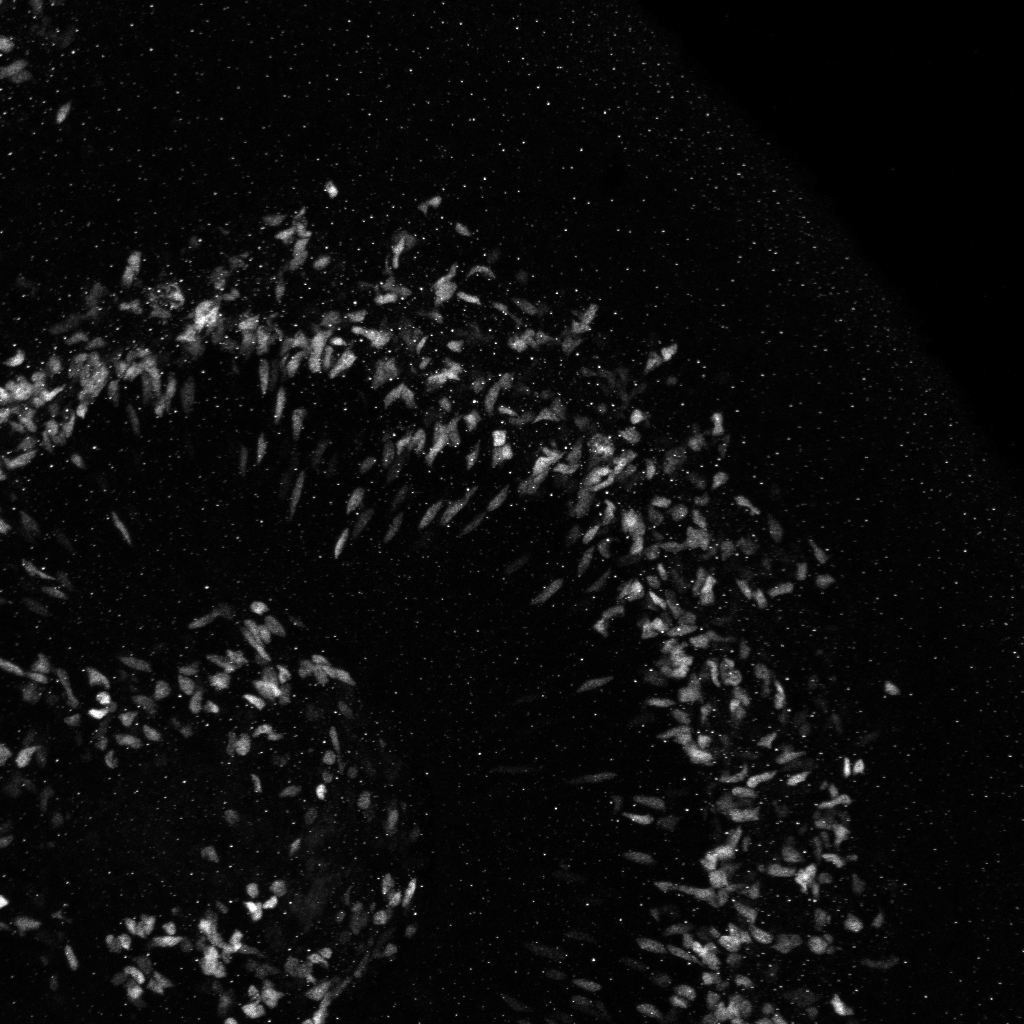

Supplement: Supplementary file 7 — Source Data Fig. 3 [file 44318_2024_68_MOESM7_ESM.zip › Figure 3/3F/Fig3F_gEREG KO1+2_DAPI, GFP(488),SOX2(555), TBR2(647).tif]

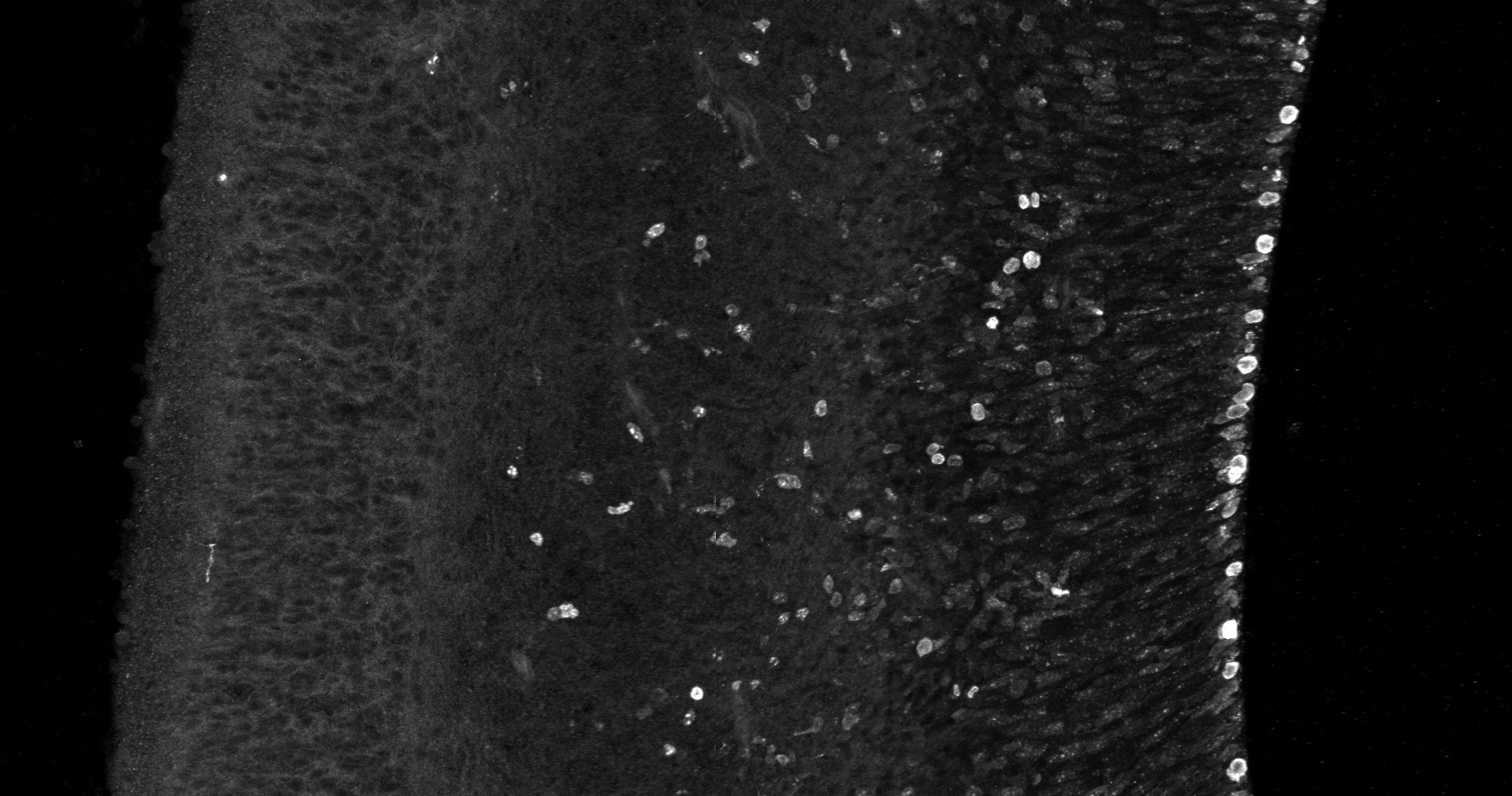

Supplement: Supplementary file 8 — Source Data Fig. 4 [file 44318_2024_68_MOESM8_ESM.zip › Figure 4/4B/Fig4B_HT_50ng_DAPI, TBR2(488),SOX2(555),KI67(647).tif]

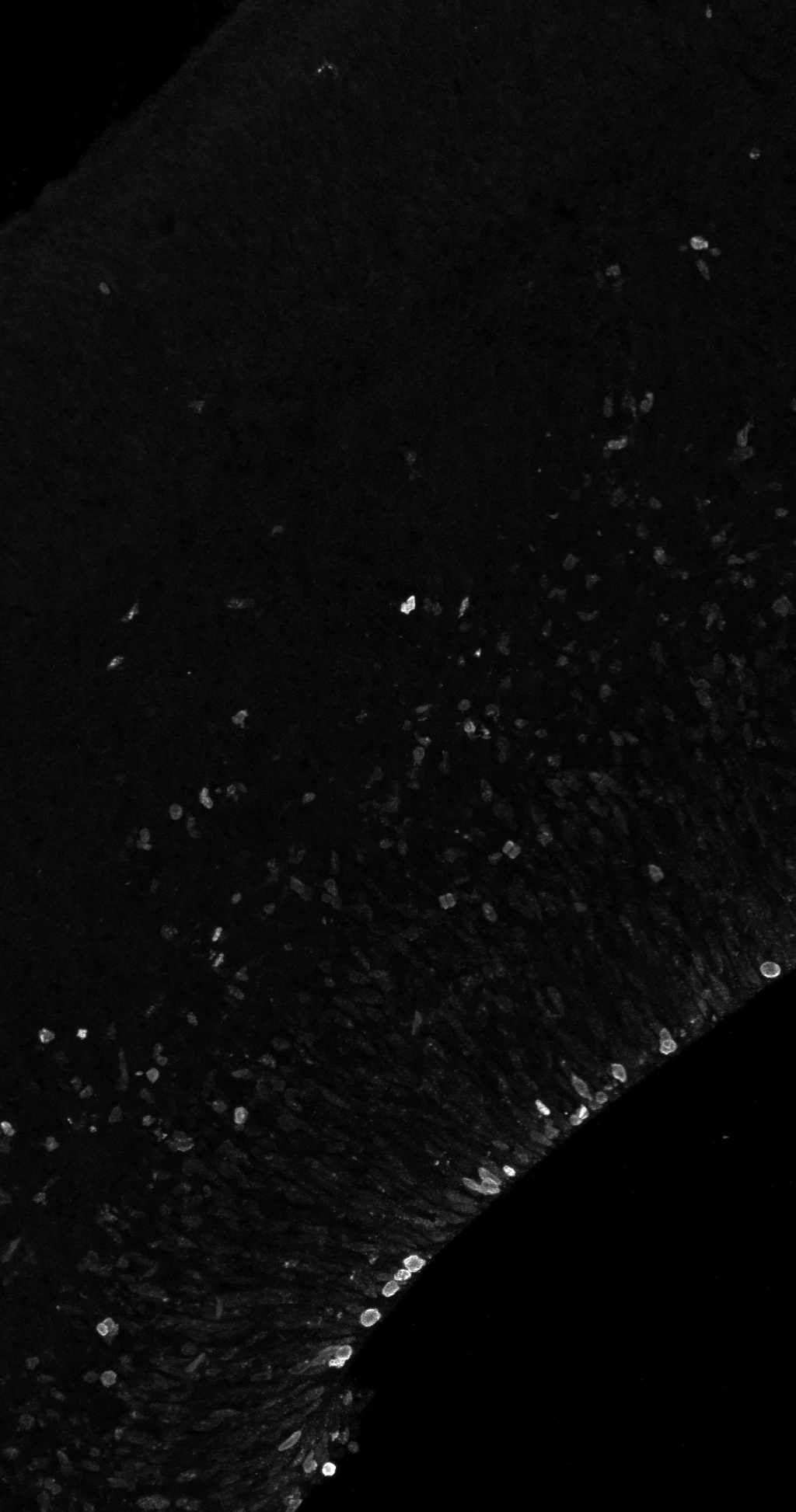

Supplement: Supplementary file 8 — Source Data Fig. 4 [file 44318_2024_68_MOESM8_ESM.zip › Figure 4/4B/Fig4B_HT_Crl_DAPI, TBR2(488),SOX2(555),KI67(647).tif]

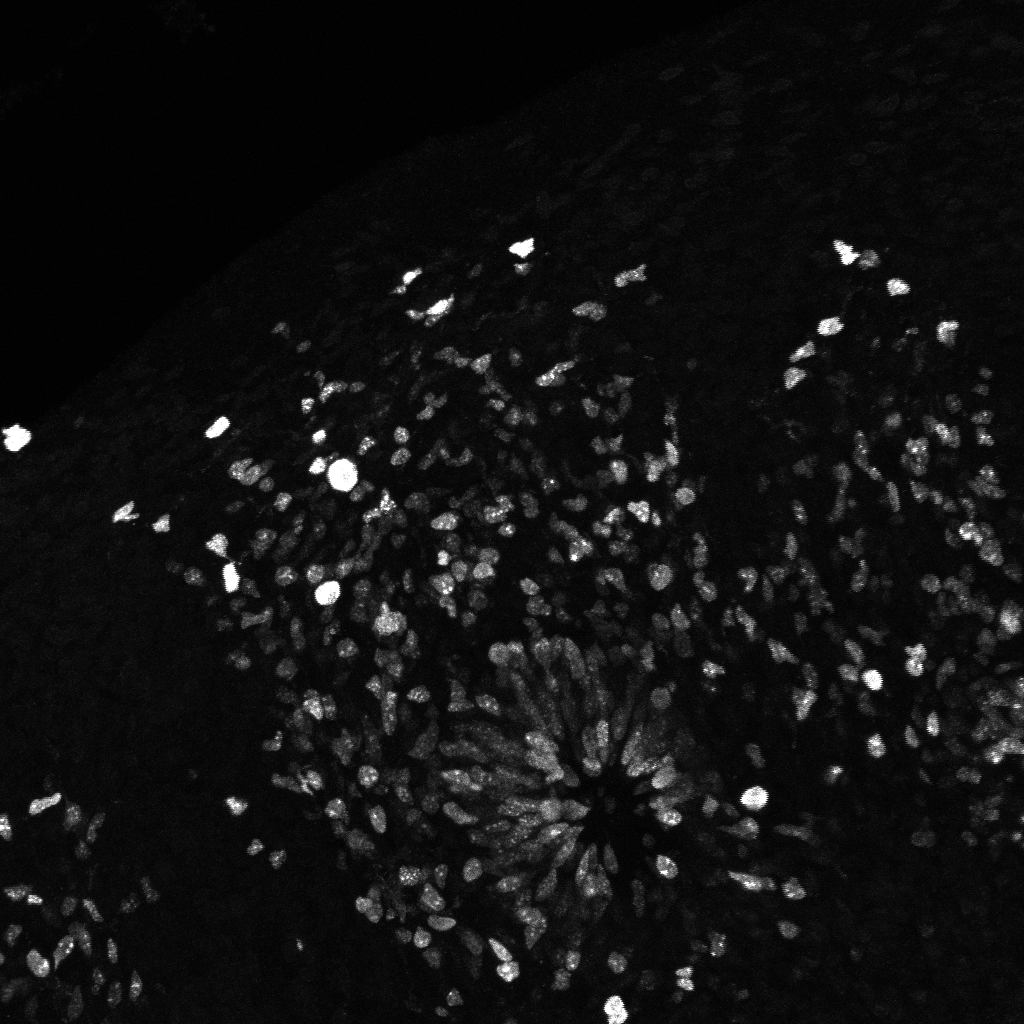

Supplement: Supplementary file 8 — Source Data Fig. 4 [file 44318_2024_68_MOESM8_ESM.zip › Figure 4/4G/Fig4G_hCO_Crl_DAPI, TBR2(488),SOX2(555),KI67(647).tif]

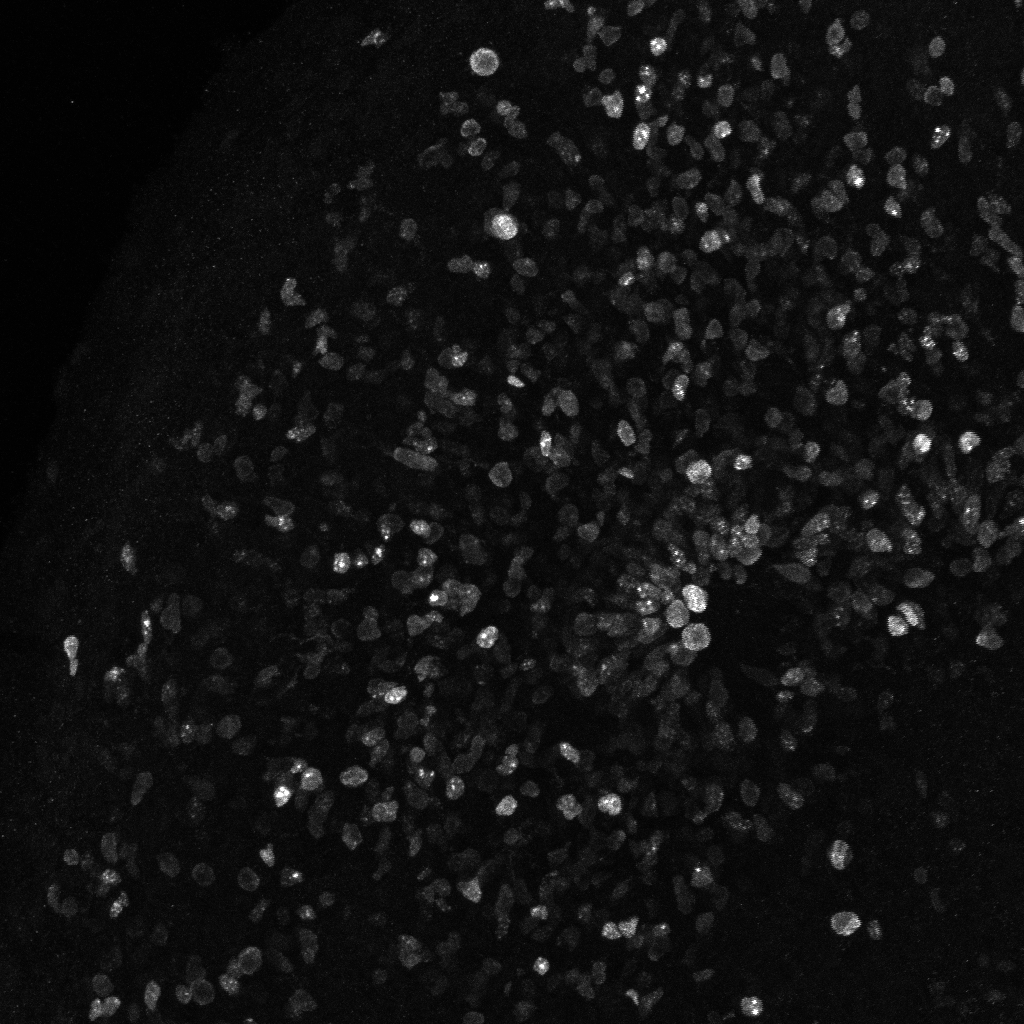

Supplement: Supplementary file 8 — Source Data Fig. 4 [file 44318_2024_68_MOESM8_ESM.zip › Figure 4/4G/Fig4G_hCO_50ng_DAPI, TBR2(488),SOX2(555),KI67(647).tif]

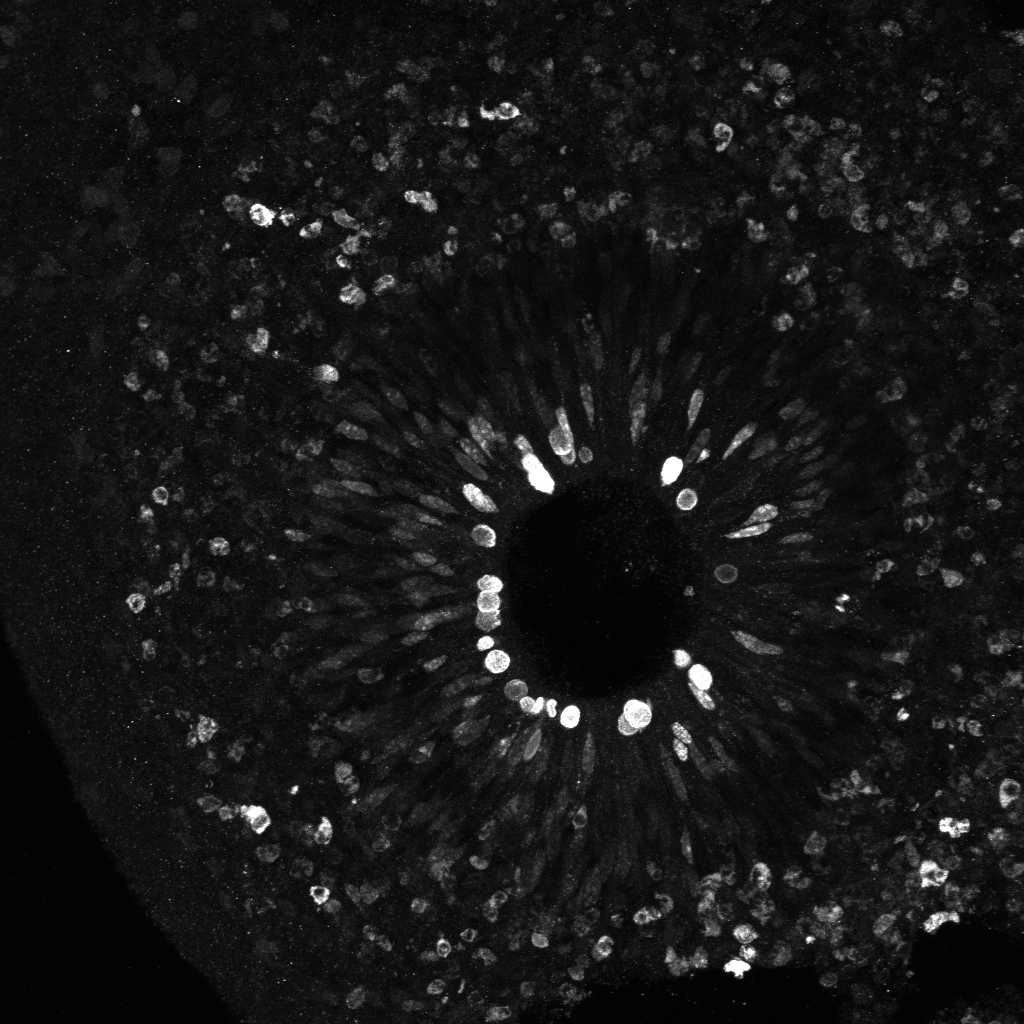

Supplement: Supplementary file 9 — Source Data Fig. 5 [file 44318_2024_68_MOESM9_ESM.zip › Figure 5/5B/Fig5B_gCO_50ng_DAPI, TBR2(488), SOX2(555), KI67(647).tif]

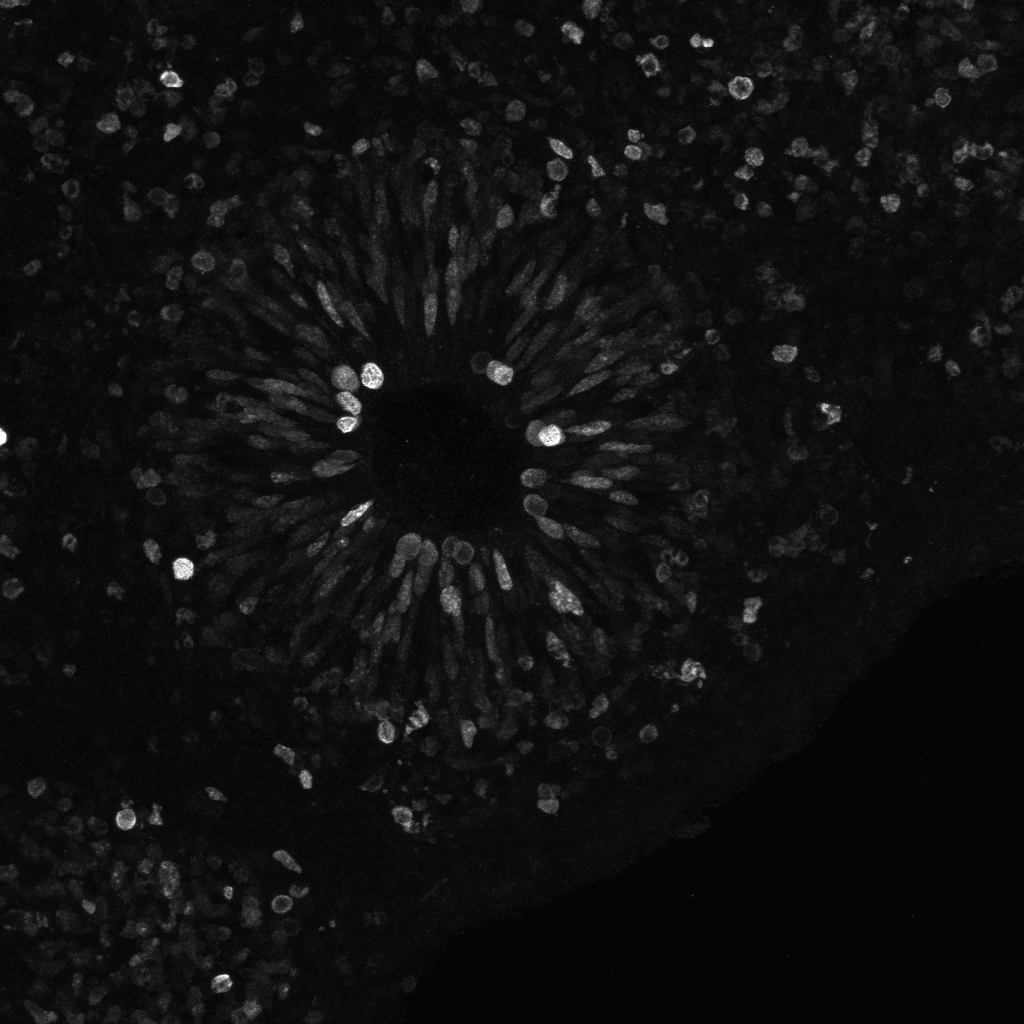

Supplement: Supplementary file 9 — Source Data Fig. 5 [file 44318_2024_68_MOESM9_ESM.zip › Figure 5/5B/Fig5B_gCO_ctrl_DAPI, TBR2(488), SOX2(555), KI67(647).tif]

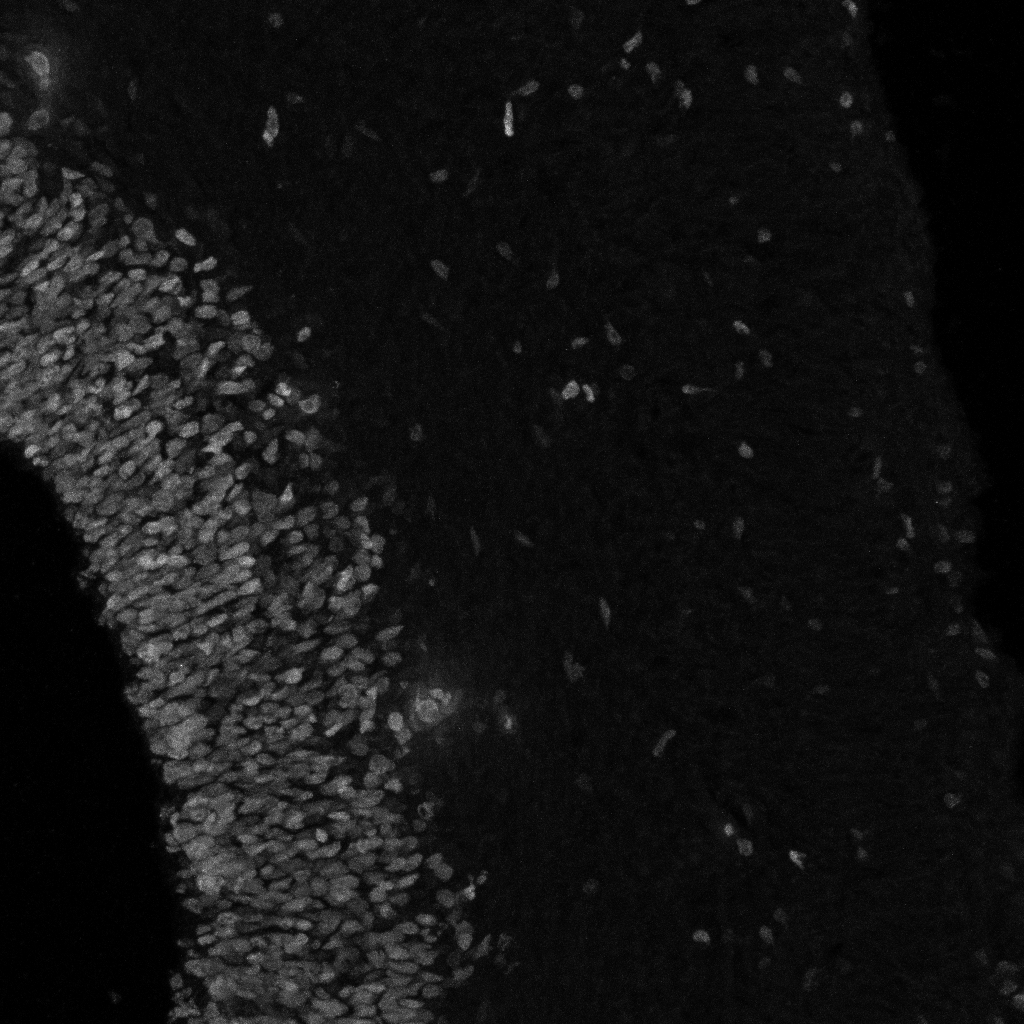

Supplement: Supplementary file 10 — Source Data Fig. 6 [file 44318_2024_68_MOESM10_ESM.zip › Figure 6/6H/Fig6H_etoh_DAPI, PH3 (488),Sox2(555).tif]

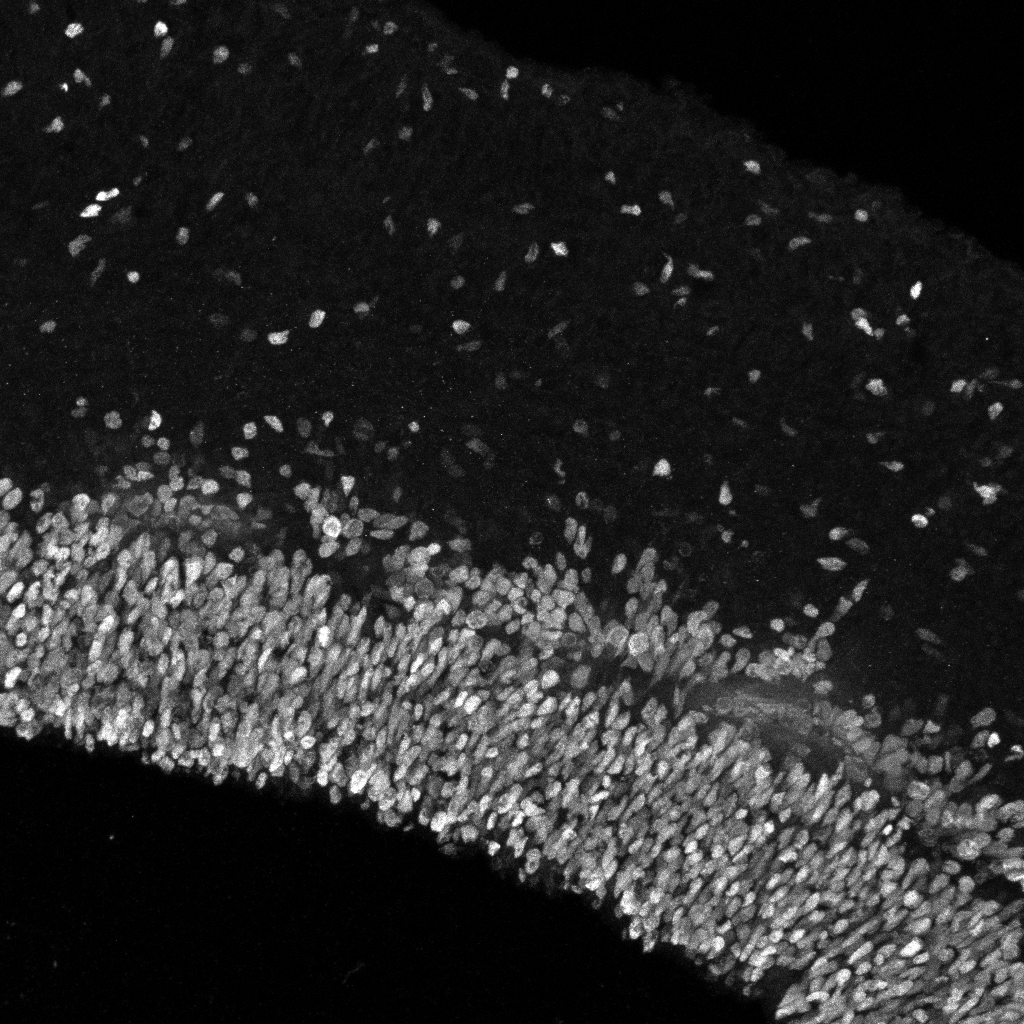

Supplement: Supplementary file 10 — Source Data Fig. 6 [file 44318_2024_68_MOESM10_ESM.zip › Figure 6/6H/Fig6H_etoh+epi_DAPI, PH3 (488),Sox2(555).tif]

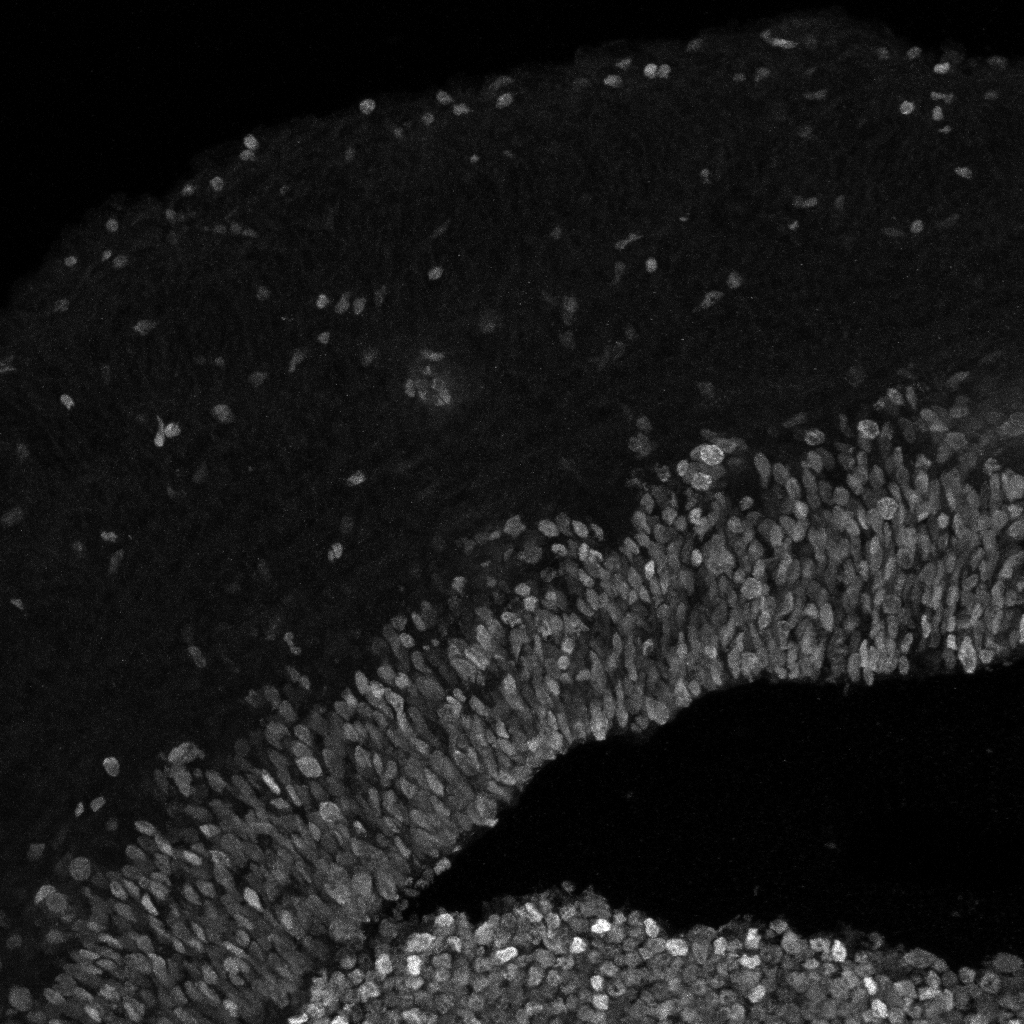

Supplement: Supplementary file 10 — Source Data Fig. 6 [file 44318_2024_68_MOESM10_ESM.zip › Figure 6/6H/Fig6H_AG1478_DAPI, PH3 (488),Sox2(555).tif]

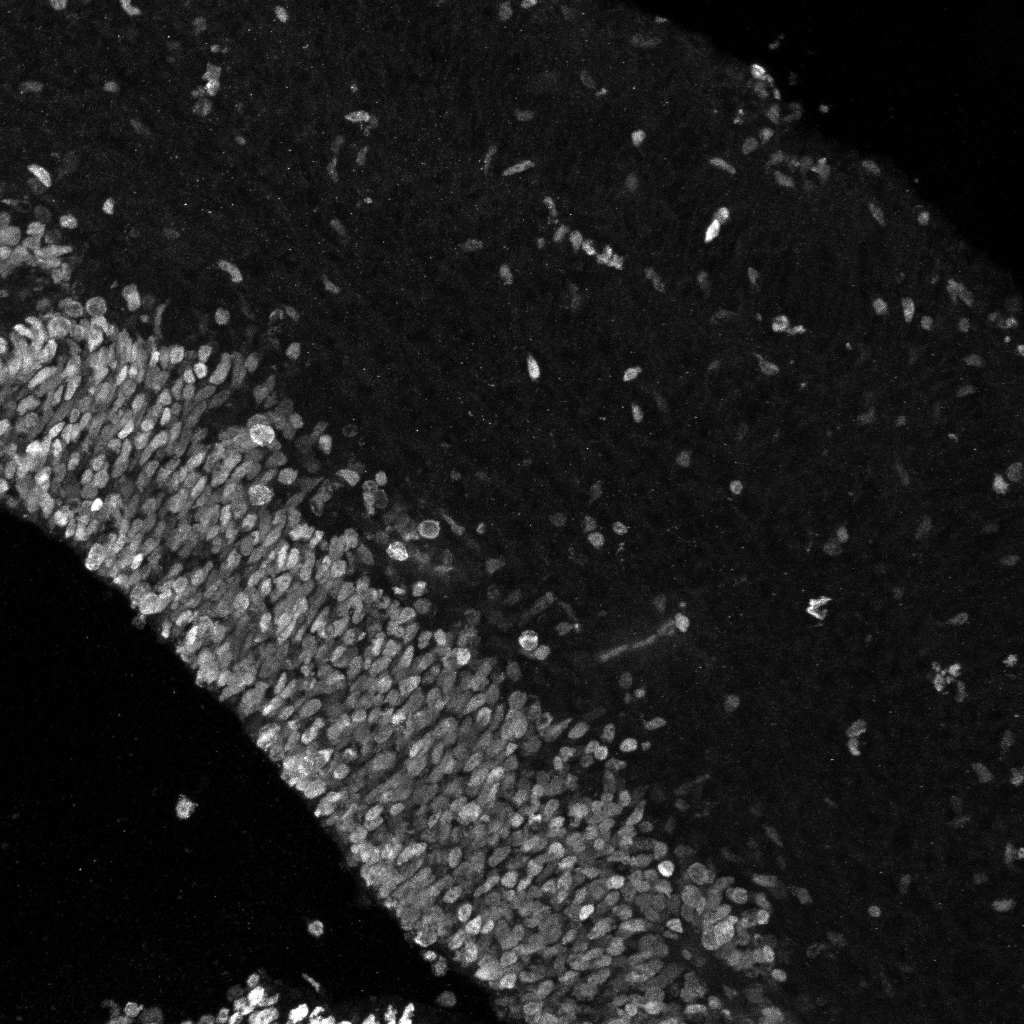

Supplement: Supplementary file 10 — Source Data Fig. 6 [file 44318_2024_68_MOESM10_ESM.zip › Figure 6/6H/Fig6H_Dacomitinib_DAPI, PH3 (488),Sox2(555).tif]

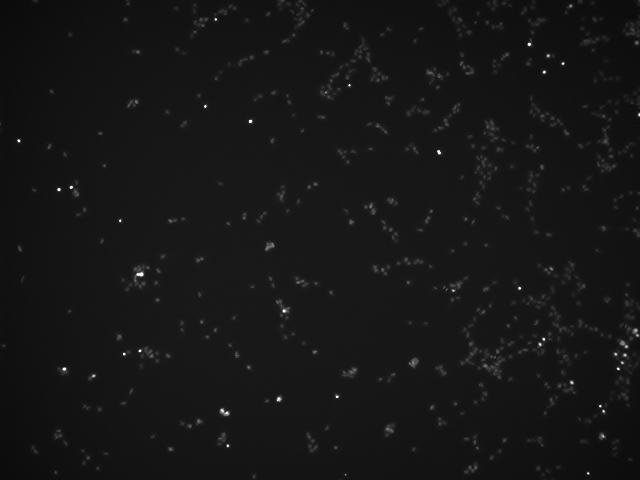

Supplement: Supplementary file 10 — Source Data Fig. 6 [file 44318_2024_68_MOESM10_ESM.zip › Figure 6/6E/Fig6E_mNSC withotEGF_day1_50ng.tif]

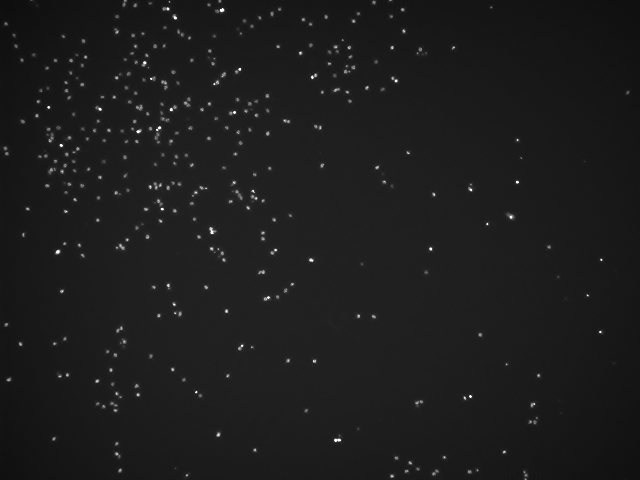

Supplement: Supplementary file 10 — Source Data Fig. 6 [file 44318_2024_68_MOESM10_ESM.zip › Figure 6/6E/Fig6E_mNSC withotEGF_day2_noEGF.tif]

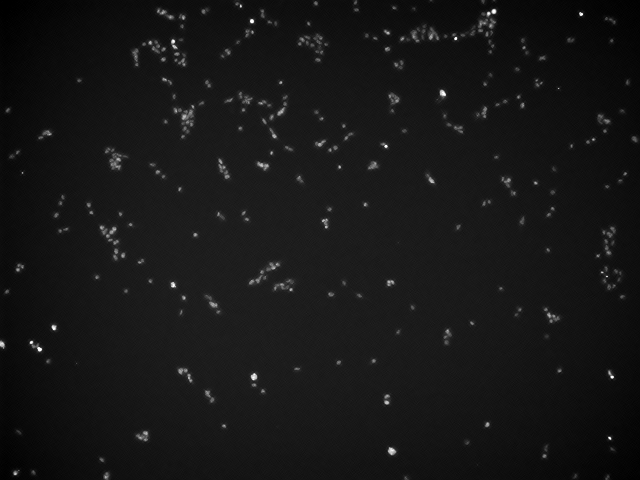

Supplement: Supplementary file 10 — Source Data Fig. 6 [file 44318_2024_68_MOESM10_ESM.zip › Figure 6/6E/Fig6E_mNSC withotEGF_day1_10ng.tif]

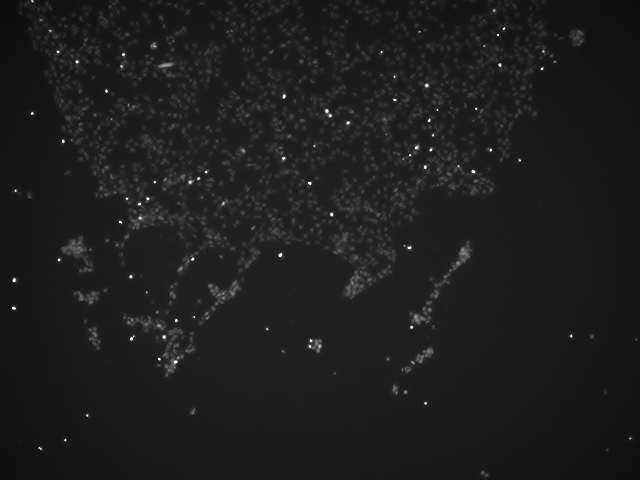

Supplement: Supplementary file 10 — Source Data Fig. 6 [file 44318_2024_68_MOESM10_ESM.zip › Figure 6/6E/Fig6E_mNSC withotEGF_day2_100ng.tif]

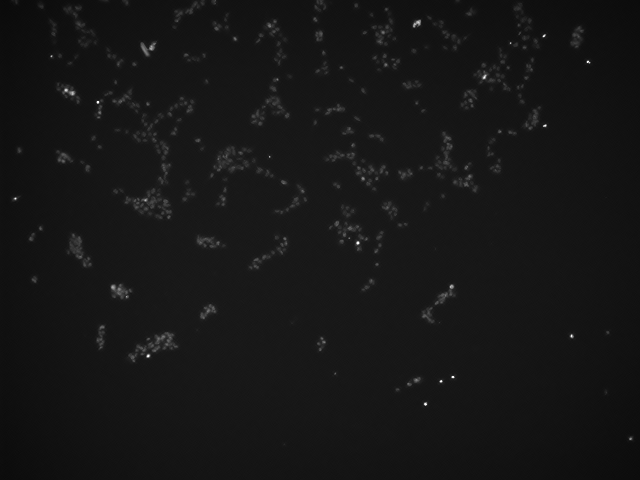

Supplement: Supplementary file 10 — Source Data Fig. 6 [file 44318_2024_68_MOESM10_ESM.zip › Figure 6/6E/Fig6E_mNSC withotEGF_day1_100ng.tif]

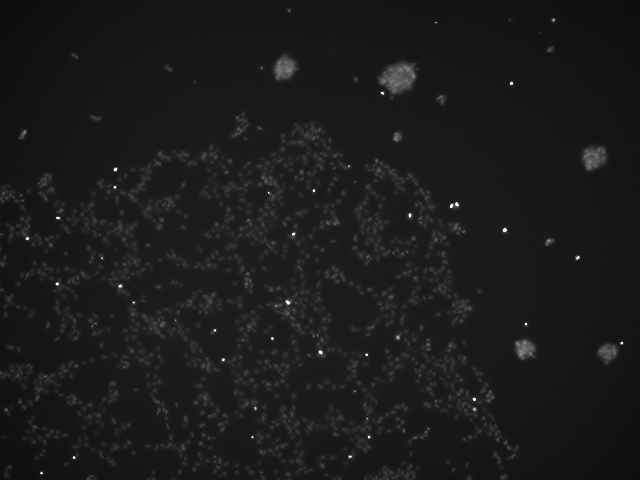

Supplement: Supplementary file 10 — Source Data Fig. 6 [file 44318_2024_68_MOESM10_ESM.zip › Figure 6/6E/Fig6E_mNSC withotEGF_day2_EGF.tif]

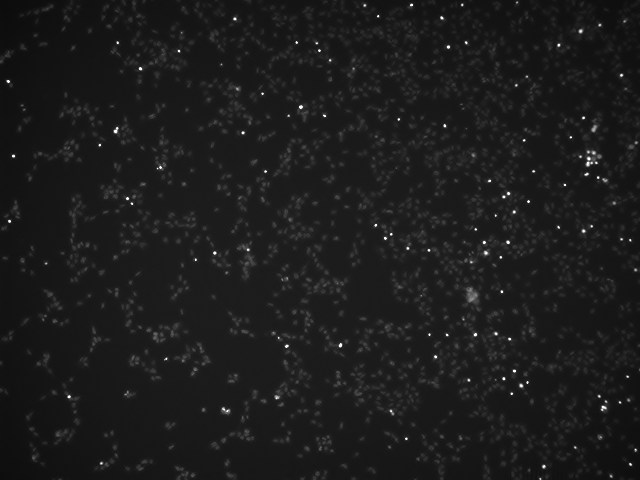

Supplement: Supplementary file 10 — Source Data Fig. 6 [file 44318_2024_68_MOESM10_ESM.zip › Figure 6/6E/Fig6E_mNSC withotEGF_day2_50ng.tif]

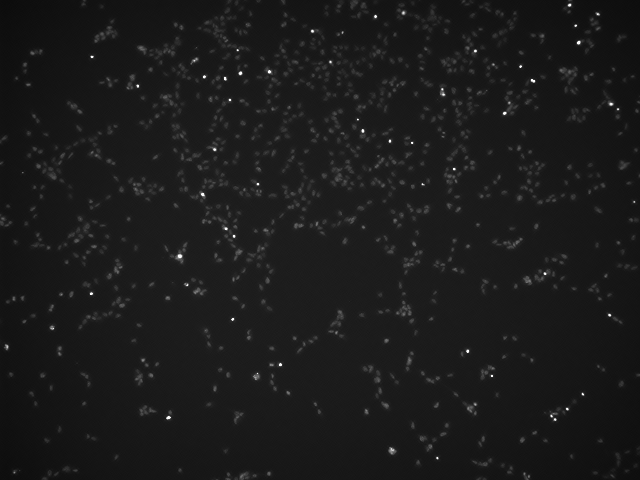

Supplement: Supplementary file 10 — Source Data Fig. 6 [file 44318_2024_68_MOESM10_ESM.zip › Figure 6/6E/Fig6E_mNSC withotEGF_day2_10ng.tif]

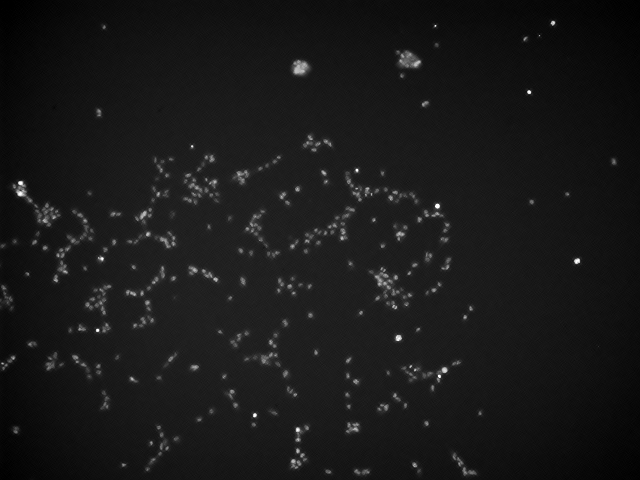

Supplement: Supplementary file 10 — Source Data Fig. 6 [file 44318_2024_68_MOESM10_ESM.zip › Figure 6/6E/Fig6E_mNSC withotEGF_day1_EGF.tif]

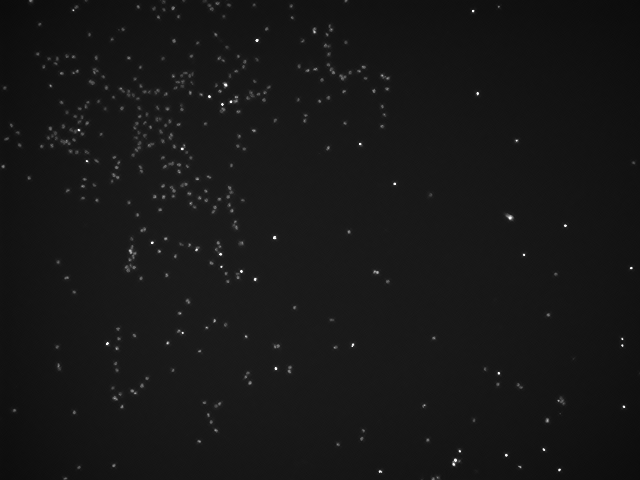

Supplement: Supplementary file 10 — Source Data Fig. 6 [file 44318_2024_68_MOESM10_ESM.zip › Figure 6/6E/Fig6E_mNSC withotEGF_day1_noEGF.tif]

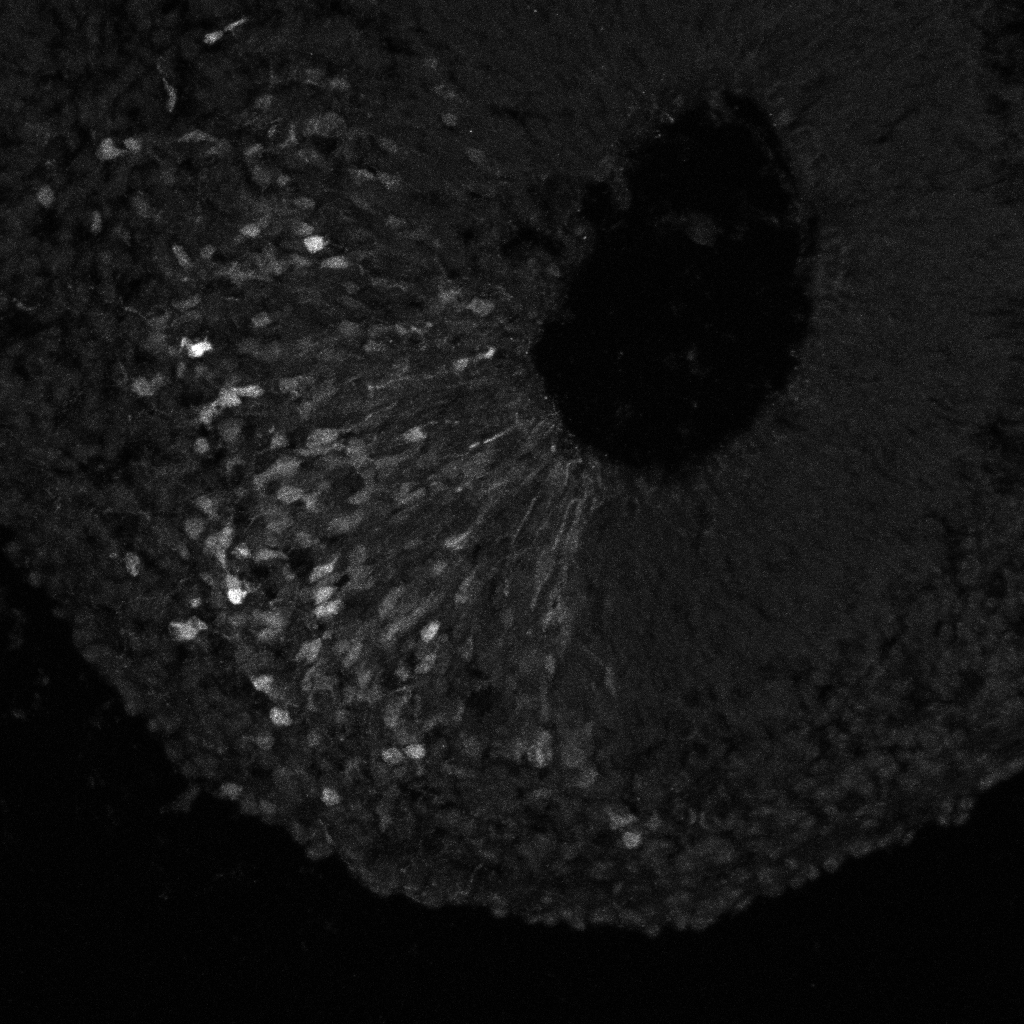

Supplement: Supplementary file 11 — Source Data Fig. 7 [file 44318_2024_68_MOESM11_ESM.zip › Figure 7/7E/Fig7E_hCRE6_DAPI, GFP(488), mScarlet (647).tif]

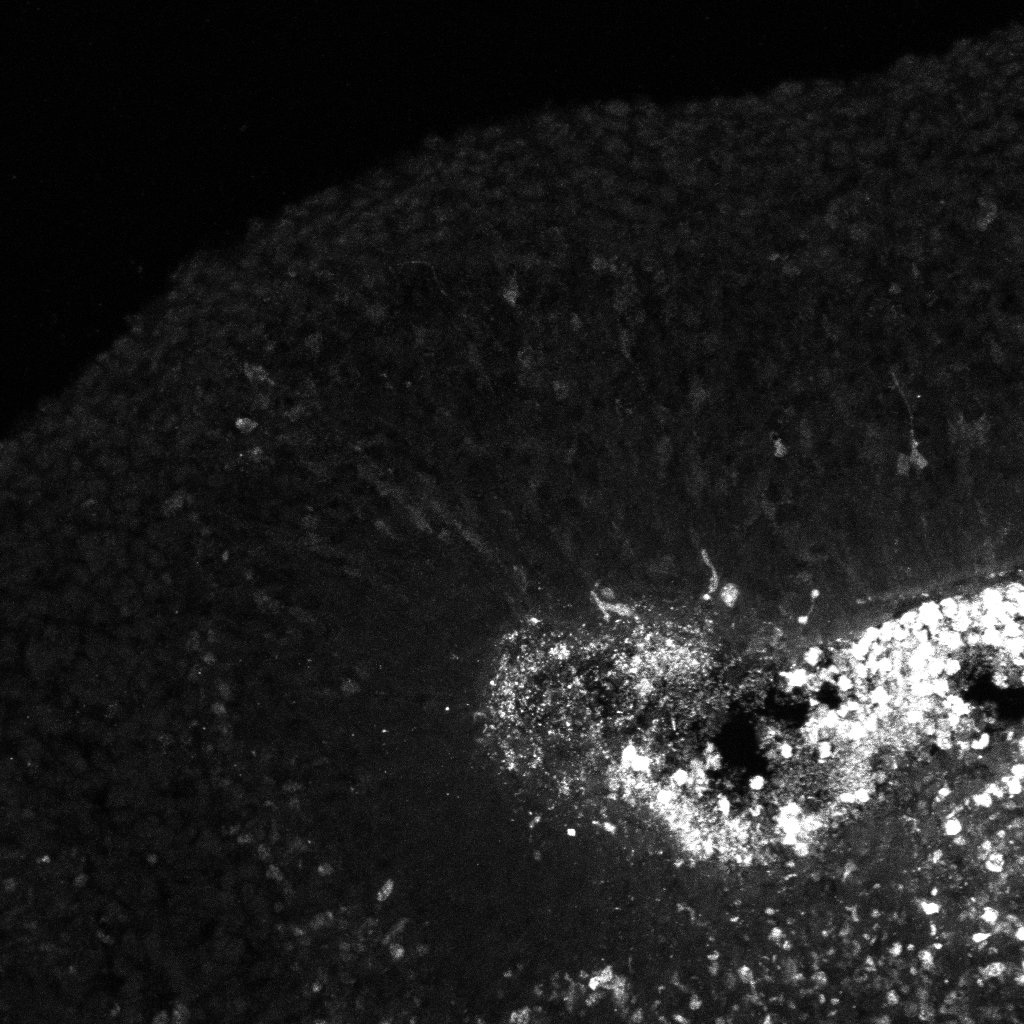

Supplement: Supplementary file 11 — Source Data Fig. 7 [file 44318_2024_68_MOESM11_ESM.zip › Figure 7/7E/Fig7E_mCRE6_DAPI, GFP(488), mScarlet (647).tif]

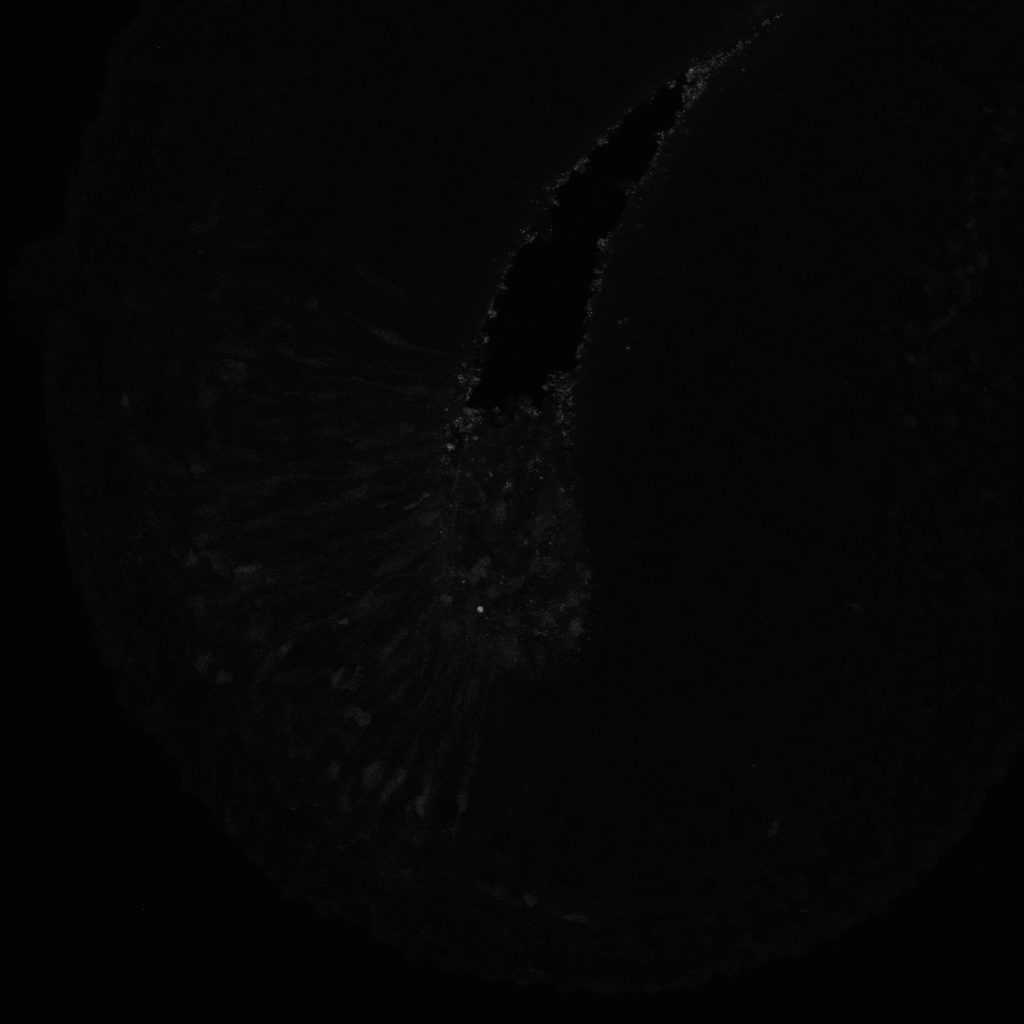

Supplement: Supplementary file 11 — Source Data Fig. 7 [file 44318_2024_68_MOESM11_ESM.zip › Figure 7/7E/Fig7E_mCRE9_DAPI, GFP(488), mScarlet (647).tif]

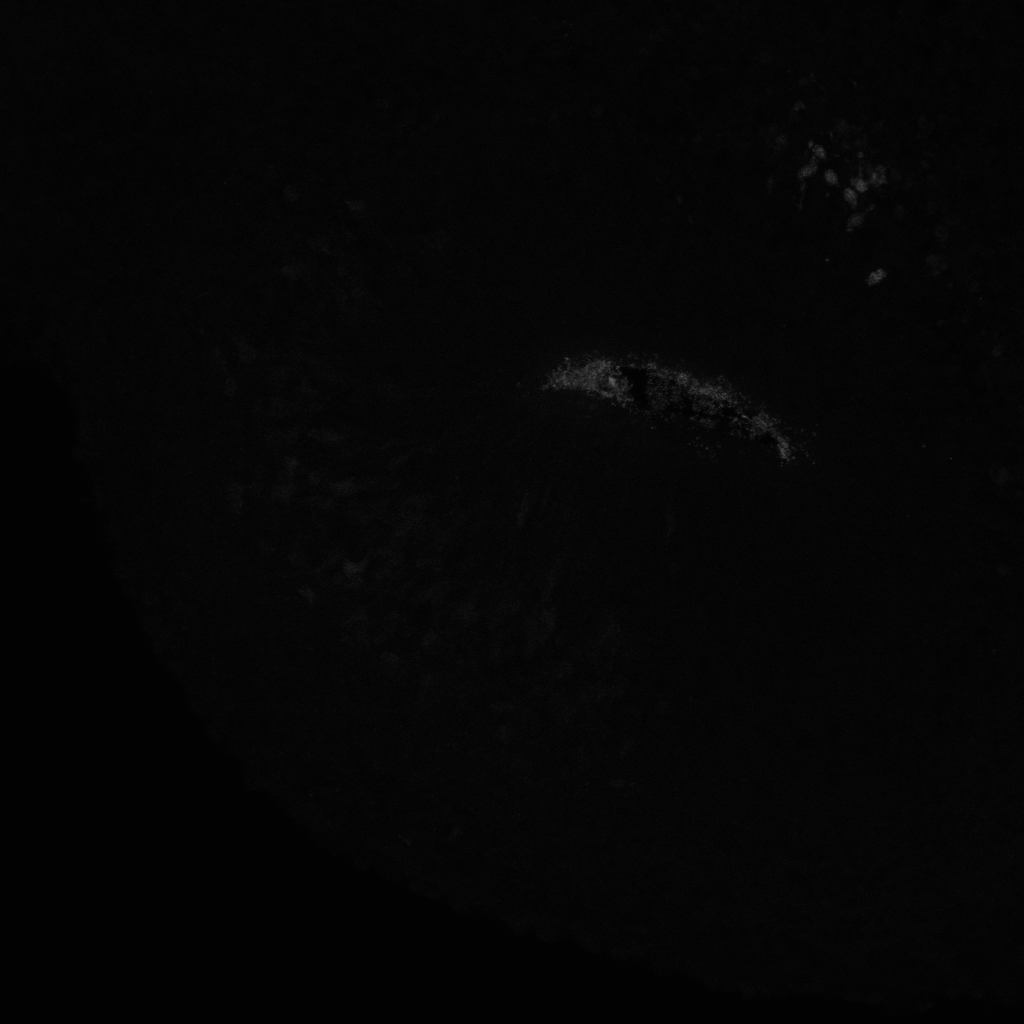

Supplement: Supplementary file 11 — Source Data Fig. 7 [file 44318_2024_68_MOESM11_ESM.zip › Figure 7/7E/Fig7E_hCRE9_DAPI, GFP(488), mScarlet (647).tif]

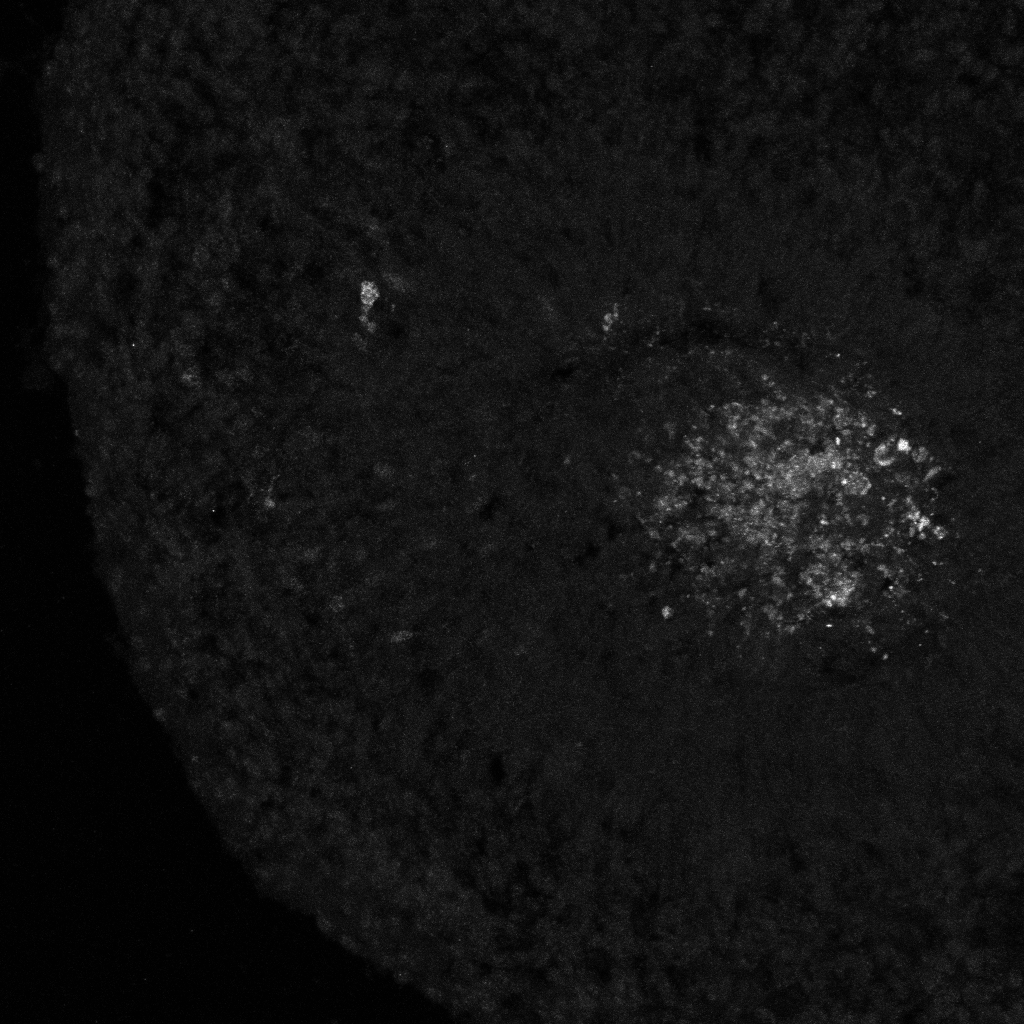

Supplement: Supplementary file 11 — Source Data Fig. 7 [file 44318_2024_68_MOESM11_ESM.zip › Figure 7/7E/Fig7E_scr_DAPI, GFP(488), mScarlet (647).tif]
