# Supplementary material for: The growth factor EPIREGULIN promotes basal progenitor cell proliferation in the developing neocortex
Source: EMBO J. 2024 Mar 21;43(8):2. doi: 10.1038/s44318-024-00068-7 (PMC11021537; doi:10.1038/s44318-024-00068-7)
Supplement: Supplementary file 13 — Expanded View Figures [file 44318_2024_68_MOESM13_ESM.pdf]

Expanded View Figures

Figure EV1. Expression of *EREG* in neural progenitor cells of different species.

(**A**, **B**) *EREG* mRNA levels in human fetal Ncx tissue and cerebral organoids analyzed by RNA-seq (data from Camp et al, (2015); Johnson et al, (2015)). (**C**) In situ hybridization data for *Sox2* and *Ereg* of E13.5 and E15.5 mouse neocortex, obtained from the Allen Brain Atlas (Allen Institute for Brain Science, 2004). (**D**) Immunofluorescence for SOX2, TBR2, and TUJ1 of mNcx and hNcx tissue. (**E**) H3K4me3, H3K27me3, and H3K27ac ChIP-seq signal around the *EREG* transcription start site (±1 kb) in mouse proliferative aRG, forebrain, and cortex (top) and of H3K27ac in the human cortex (bottom) (data from Albert and Huttner (2018); Gorkin et al, (2020); Reilly et al, (2015)). (**F**) Immunofluorescence for SOX2, TBR2, and TUJ1 of gorilla cerebral and human cortical organoids. (**G**) *EREG* mRNA levels in macaque and human NPCs analyzed by RNA-seq (data from Kliesmete et al, (2023)). (**H**) *EREG* mRNA levels in the ferret Ncx analyzed by microarray (data from de Juan Romero et al, (2015)). Data information: Scale bars, 100 µm. Bar graphs represent mean values. Error bars represent SD; **G**, of three samples; **H**, of six micro-dissected tissue samples.

© The Author(s)

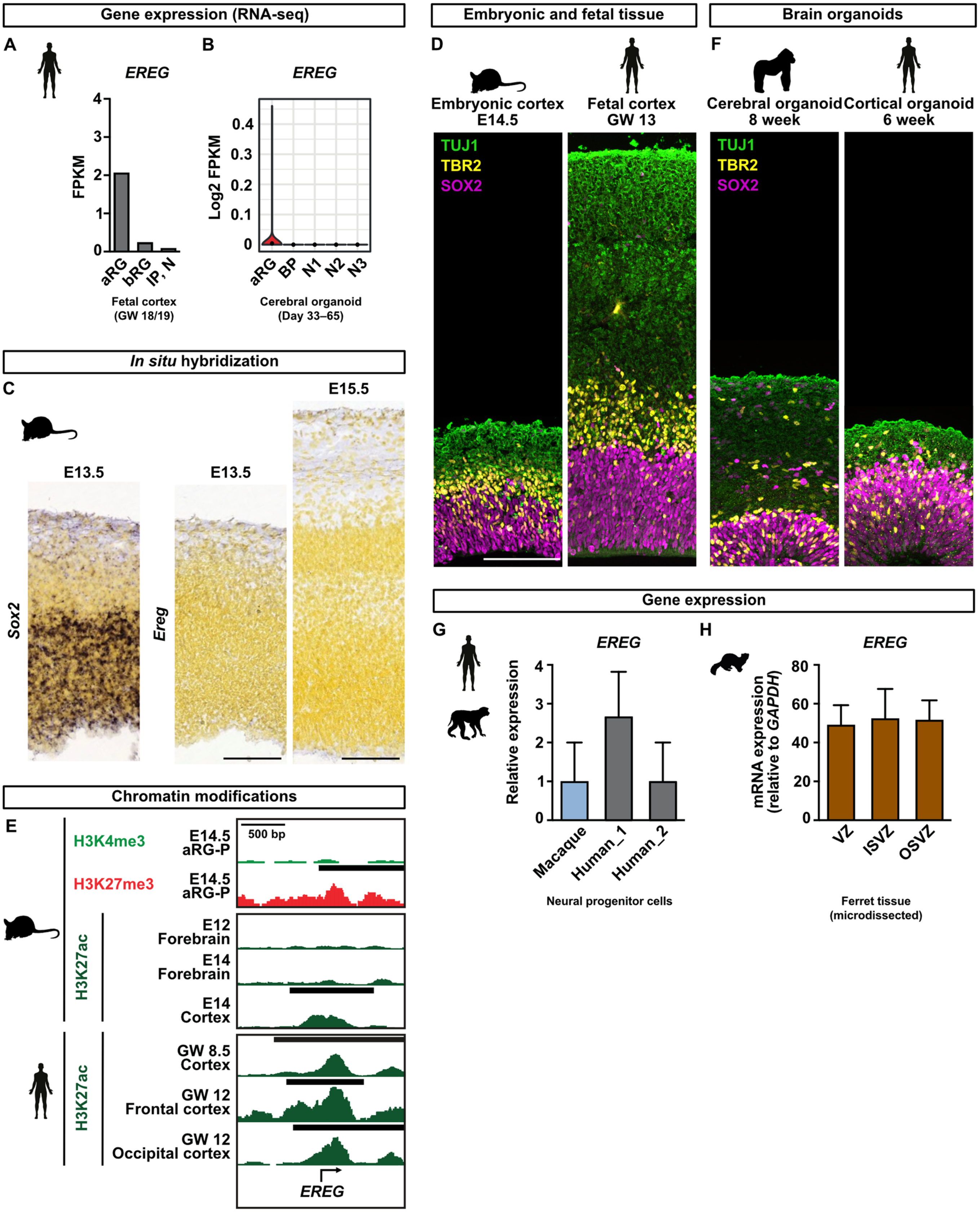
Gene expression (RNA-seq)
A
EREG
FPKM
aRG
bRG
IP, N
Fetal cortex (GW 18/19)
B
EREG
Log2 FPKM
aRG
BP
N1
N2
N3
Cerebral organoid (Day 33–65)
In situ hybridization
C
E13.5
E13.5
E15.5
Sox2
Ereg
Chromatin modifications
E
H3K4me3
E14.5 aRG-P
H3K27me3
E14.5 aRG-P
500 bp
H3K27ac
E12 Forebrain
E14 Forebrain
E14 Cortex
H3K27ac
GW 8.5 Cortex
GW 12 Frontal cortex
GW 12 Occipital cortex
EREG
Embryonic and fetal tissue
D
Embryonic cortex E14.5
Fetal cortex GW 13
TUJ1
TBR2
SOX2
Brain organoids
F
Cerebral organoid 8 week
Cortical organoid 6 week
TUJ1
TBR2
SOX2
Gene expression
G
EREG
Relative expression
Macaque
Human_1
Human_2
Neural progenitor cells
H
EREG
mRNA expression (relative to GAPDH)
VZ
ISVZ
OSVZ
Ferret tissue (microdissected)

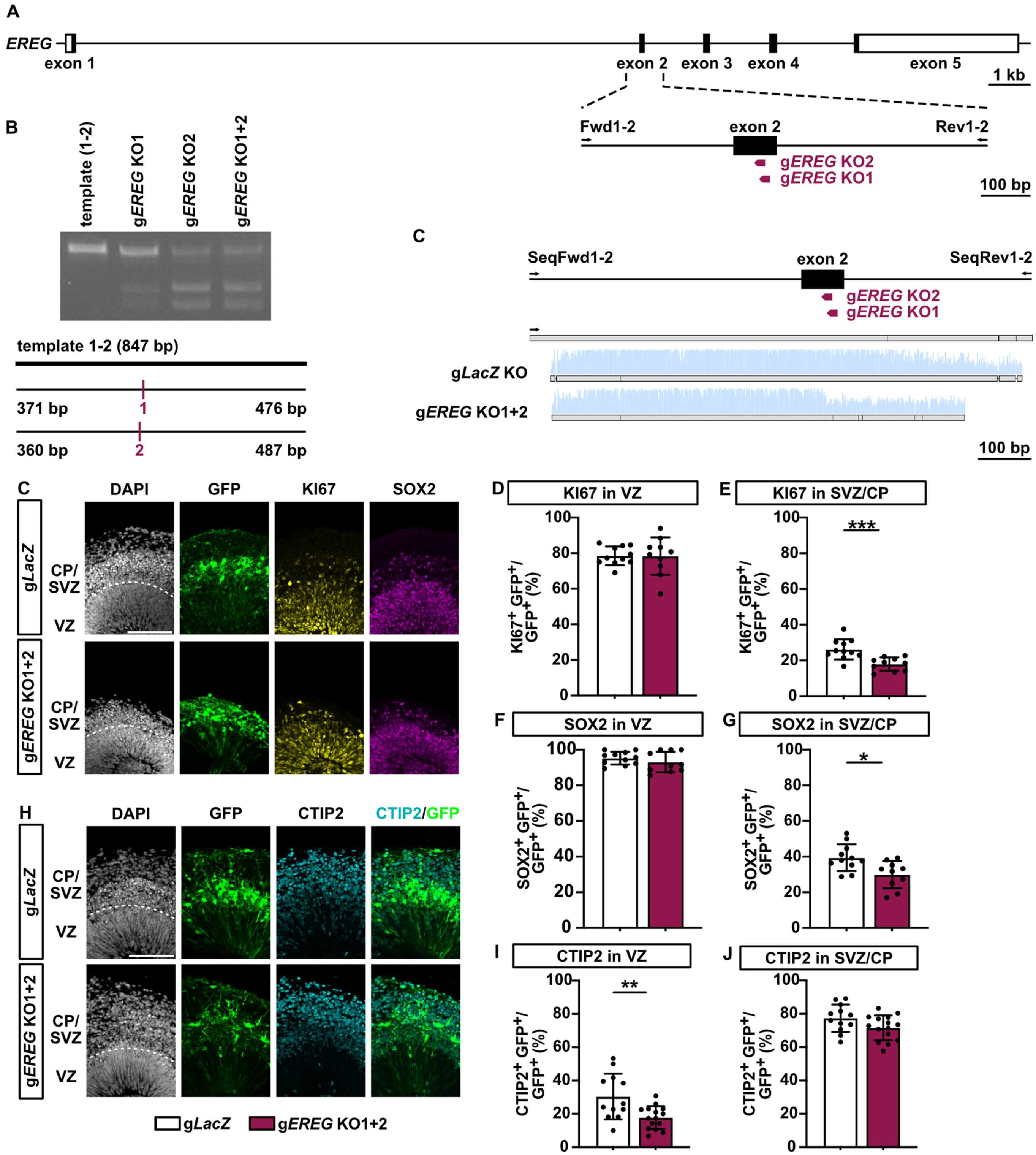
A
EREG
exon 1
exon 2
exon 3
exon 4
exon 5
1 kb
Fwd1-2
exon 2
Rev1-2
gEREG KO2
gEREG KO1
100 bp
B
template (1-2)
gEREG KO1
gEREG KO2
gEREG KO1+2
template 1-2 (847 bp)
371 bp
1
476 bp
360 bp
2
487 bp
C
SeqFwd1-2
exon 2
SeqRev1-2
gEREG KO2
gEREG KO1
gLacZ KO
gEREG KO1+2
100 bp
C
DAPI
GFP
KI67
SOX2
gLacZ
CP/
SVZ
VZ
gEREG KO1+2
D
KI67 in VZ
KI67+ GFP+/ GFP+ (%)
E
KI67 in SVZ/CP
***
F
SOX2 in VZ
SOX2+ GFP+/ GFP+ (%)
G
SOX2 in SVZ/CP
*
H
DAPI
GFP
CTIP2
CTIP2/GFP
I
CTIP2 in VZ
**
CTIP2+ GFP+/ GFP+ (%)
J
CTIP2 in SVZ/CP
gLacZ
gEREG KO1+2

 © The Author(s)

Figure EV2. Validation of *EREG* gRNA function.

(**A**) Schematic illustration of the human *EREG* gene locus. The location of the guide RNAs for CRISPR/Cas9-mediated ablation of EPIREGULIN expression (g*EREG* KO1 + 2) is shown, as well as the location of primer binding sites (Fwd, forward; Rev, reverse) for the generation of DNA templates for in vitro gRNA efficiency testing. (**B**) Guide RNA efficiencies were tested in vitro. The effects of the g*EREG* KO1 + 2 RNAs to direct Cas9-mediated cutting of PCR templates was analyzed by agarose gel electrophoresis. Schemes of the sizes of PCR templates, guide RNA binding sites, and expected sizes of cut fragments are indicated below. (**C**) CRISPR/Cas9-mediated targeting of *EREG* was confirmed in the CRTDi004-A iPSC line by electroporation of Cas9/gRNA ribonucleoprotein complexes together with a GFP plasmid, followed by FACS of GFP-positive cells, PCR amplification of the target region and Sanger sequencing. The sequencing results are shown for g*EREG* KO1 + 2. (**D**) DAPI staining and immunofluorescence for GFP, KI67, and SOX2 of an electroporated human cortical organoid derived from the HPSI0114i-kolf_2 iPSC line. (**E–H**) Quantifications of KI67 and SOX2 in the VZ and SVZ/CP. (**I**) DAPI staining and immunofluorescence for GFP and CTIP2 of an electroporated human cortical organoid from the CRTDi004-A iPSC line. (**J**, **K**) Quantifications of CTIP2 in the VZ and SVZ/CP. Data information: Scale bar, 100 µm. Bar graphs represent mean values. Error bars represent SD of 12–15 organoids from three batches. $^{**}p < 0.01$; Student's *t*-test.

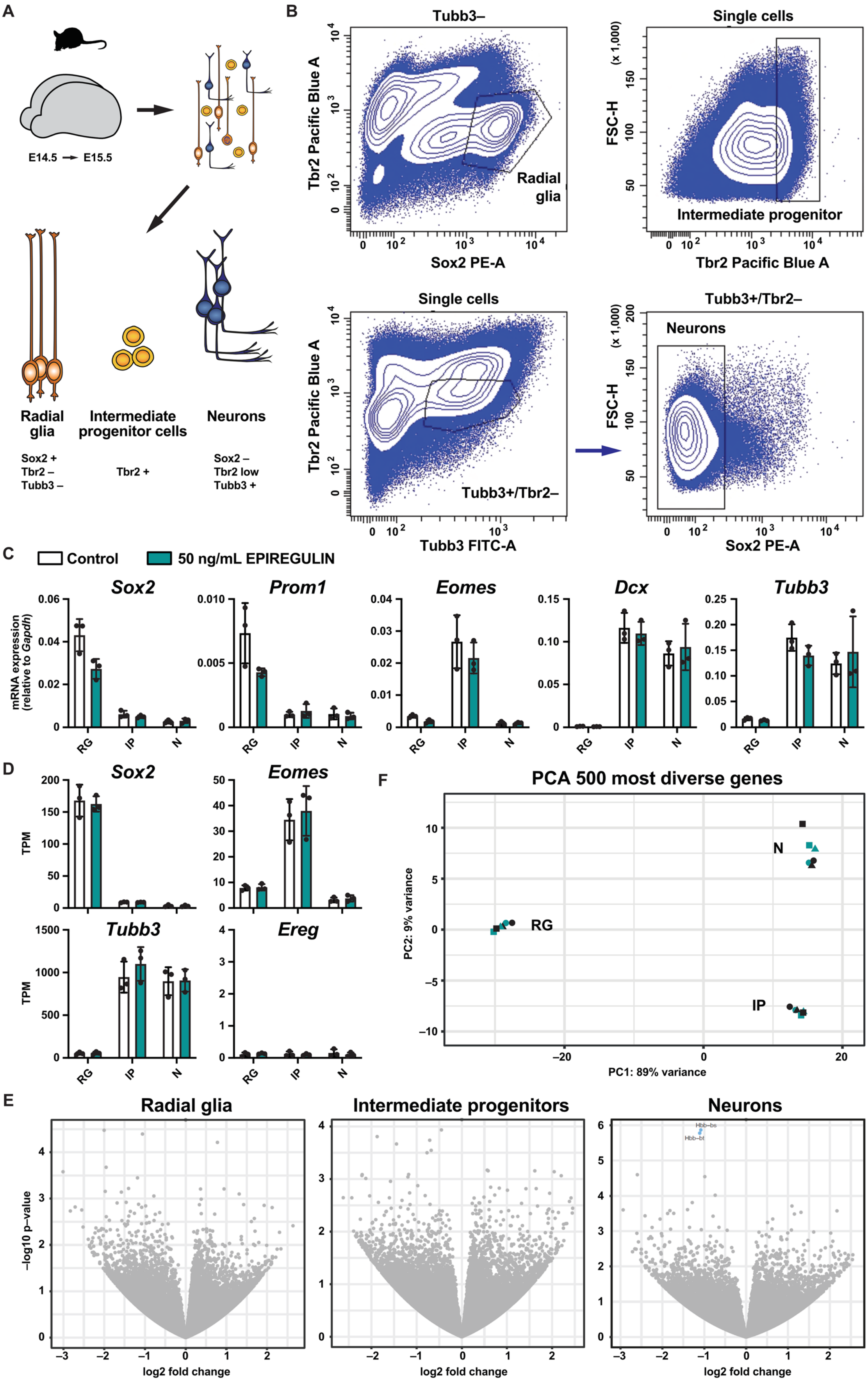
A
E14.5 → E15.5
Radial glia
Intermediate progenitor cells
Neurons
Sox2 +
Tbr2 –
Tubb3 –
Tbr2 +
Sox2 –
Tbr2 low
Tubb3 +
B
Tubb3–
Single cells
Radial glia
Intermediate progenitor
Tubb3+/Tbr2–
Neurons
Tbr2 Pacific Blue A
Sox2 PE-A
FSC-H
Tubb3 FITC-A
C
Control
50 ng/mL EPIREGULIN
Sox2
Prom1
Eomes
Dcx
Tubb3
mRNA expression (relative to Gapdh)
RG
IP
N
D
TPM
Ereg
F
PCA 500 most diverse genes
PC1: 89% variance
PC2: 9% variance
E
Radial glia
Intermediate progenitors
Neurons
–log10 p-value
log2 fold change
Hbb-bs
Hbb-bt

 © The Author(s)

Figure EV3. Gene expression analysis upon addition of EPIREGULIN to the mouse neocortex.

(**A**) Schematic illustration of the experimental workflow. Mouse brain hemispheres (E14.5) from the *Tubb3*::GPF line (Attardo et al, 2008) were isolated and cultured under rotation in the presence of 50 ng/mL of EPIREGULIN for 24 h, dissociated, stained for Sox2 and Tbr2, and cell populations isolated by immuno-FACS based on the indicated marker combinations. (**B**) Gating strategy of RG (top, left) based on high levels of Sox2 and low levels of Tbr2; IP (top, right) based on high levels of Tbr2, irrespective of other markers; and neurons (bottom) based on enrichment of GFP expressed from the *Tubb3* promoter and low level of Tbr2, followed by exclusion of Sox2-positive cells. (**C**) Confirmation of cell type identity by RT-qPCR expression analysis of marker genes characteristic of RG (*Sox2*, *Prom1*), IP (*Eomes*), and neurons (*Dcx*, *Tubb3*) for control and hemispheres treated with EPIREGULIN for 24 h relative to *Gapdh*. (**D**) Expression of *Sox2*, *Eomes*, *Tubb3*, and *Ereg* in RG, IP, and neurons analyzed by RNA-seq. (**E**) Volcano plots of log10 (*p* value) against log2 fold change representing the differences in gene expression in the indicated cell types analyzed by RNA-seq. Gray, non-significant; blue, downregulated. (**F**) Principal component analysis (PCA) based on the 500 most divergent genes. The percentage of variance covered by the first two components is indicated. Data information: Bar graphs represent mean values. Error bars represent SD of 3 mNcx samples from different litters. **E**, Wald test of DESeq2 was used and *P* Values were corrected for multiple testing with the Independent Hypothesis Weighting package (IHW 1.18.0).

© The Author(s)

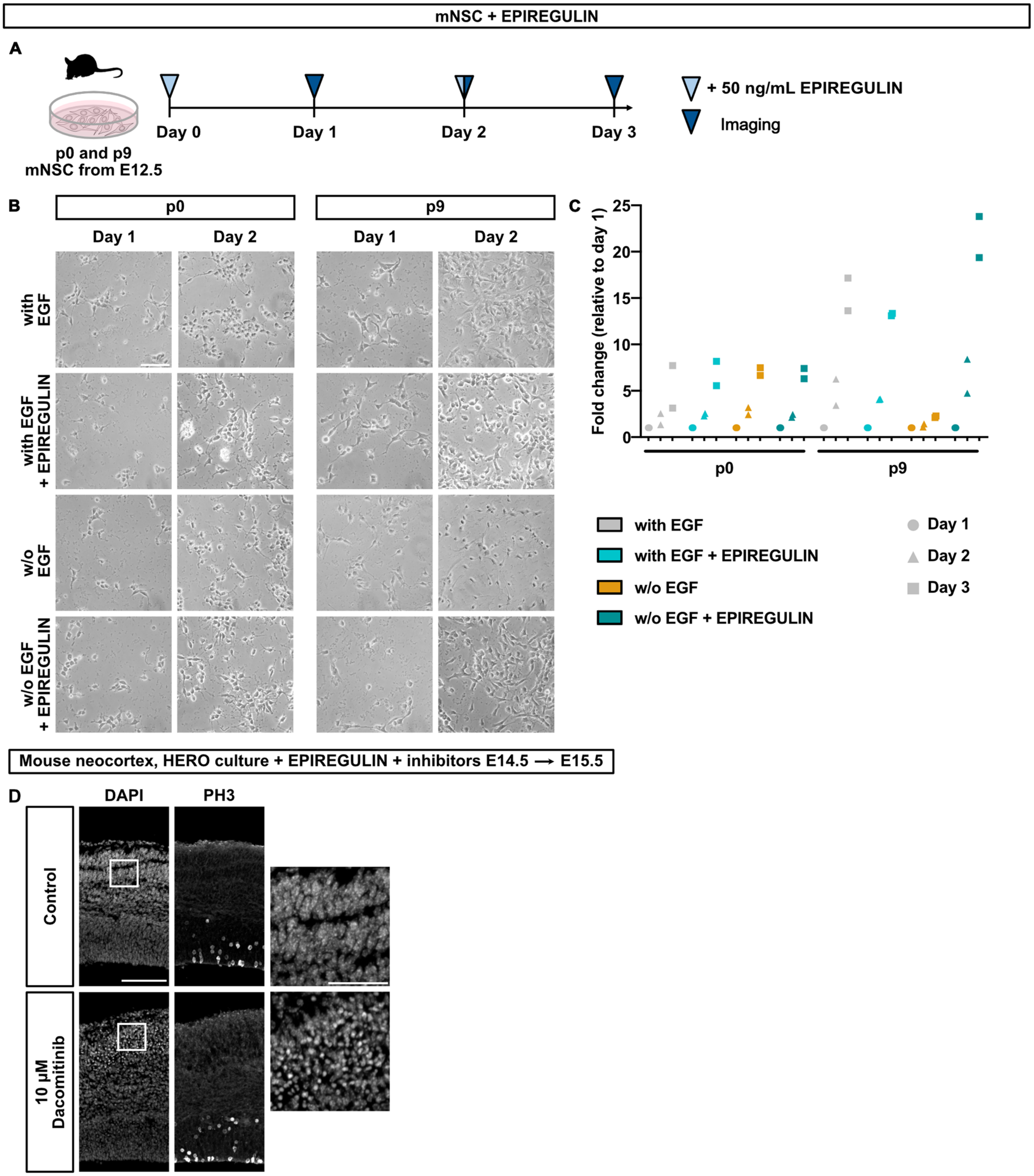
mNSC + EPIREGULIN
A
p0 and p9
mNSC from E12.5
Day 0
Day 1
Day 2
Day 3
+ 50 ng/mL EPIREGULIN
Imaging
B
p0
p9
Day 1
Day 2
with EGF
with EGF + EPIREGULIN
w/o EGF
w/o EGF + EPIREGULIN
C
Fold change (relative to day 1)
0
5
10
15
20
25
with EGF
with EGF + EPIREGULIN
w/o EGF
w/o EGF + EPIREGULIN
Day 1
Day 2
Day 3
Mouse neocortex, HERO culture + EPIREGULIN + inhibitors E14.5 ⟶ E15.5
D
DAPI
PH3
Control
10 μM Dacomitinib

 © The Author(s)

Figure EV4. NSC proliferation upon exposure to different growth factors.

(**A**) Schematic illustration of the experimental workflow. Early (p0) and late (p9) passage mouse NSC cultures were treated with different growth factors for 3 days and their proliferation was assessed. (**B**) Images of mNSC cultures following treatment with EPIREGULIN in culture medium containing FGF and with or without EGF. The control mNSCs were cultured in a medium with EGF and FGF. (**C**) Quantification of cells on days 1, 2, and 3 following EPIREGULIN treatment, either with or without EGF, shown as fold change relative to 1 h. (**D**) Staining for DAPI and immunofluorescence for PH3 of mNcx slices treated with 10 μM of the receptor inhibitor Dacomitinib for 24 h. Note the reduced tissue integrity and the apoptotic nuclei in the inset (right). Data information: Scale bars, 100 μm. **C**, Data points are from two different mNSC lines.

© The Author(s)

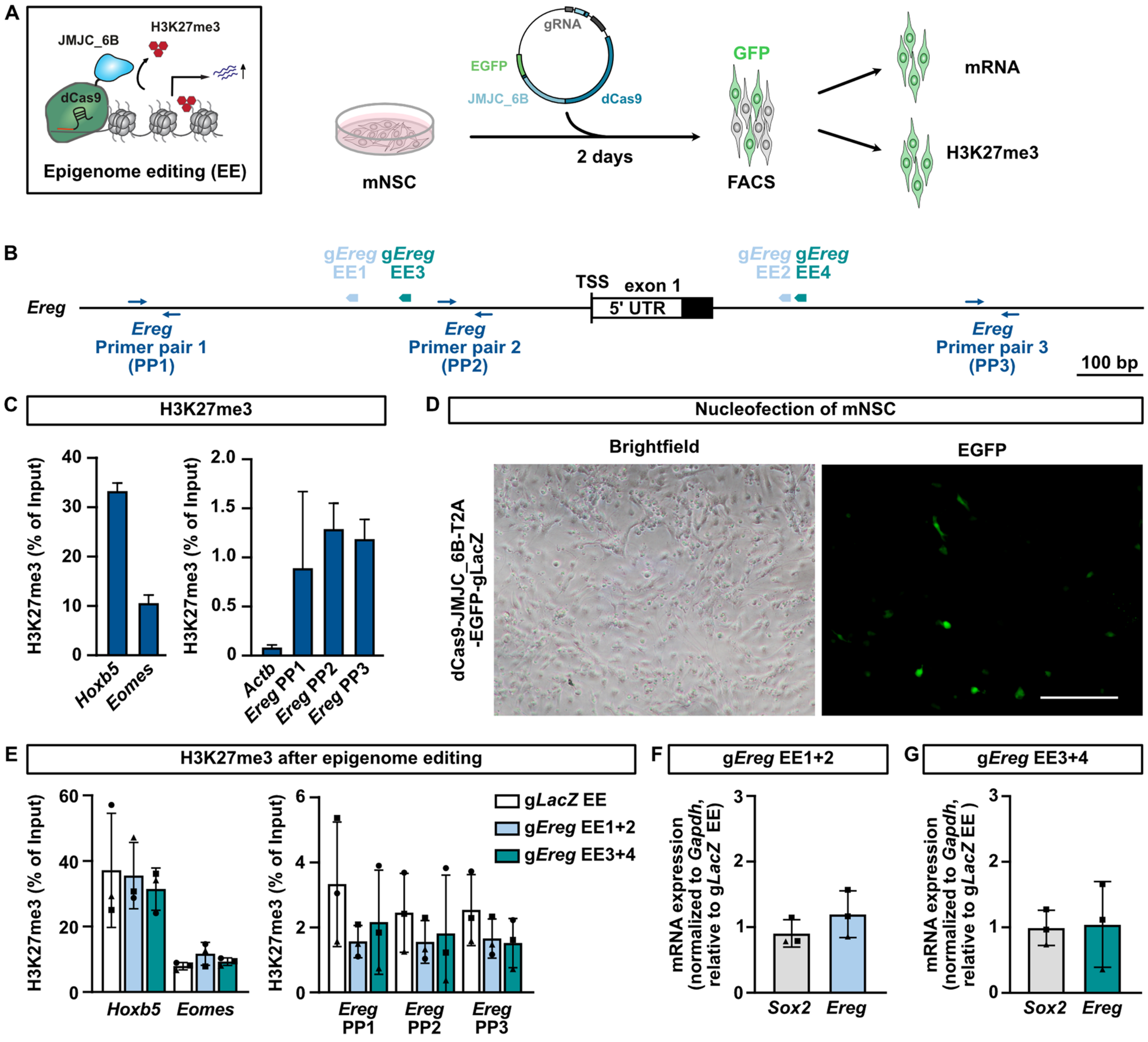

Figure EV5. Editing of histone methylation at the *Ereg* locus in mNSCs.

(**A**) Epigenome editing (EE) employing the catalytic domain of KDM6B (JMJC_6B) fused to nuclease deficient Cas9 (dCas9) in mNSCs. Histone methylation and gene expression were analyzed 2 days post-nucleofection following FACS isolation of GFP-positive cells. (**B**) The location of the gRNAs and primer binding sites (PP, primer pair) for ChIP-qPCR is shown for the *Ereg* locus. Guide RNAs g*Ereg* EE1 + 2 and g*Ereg* EE3 + 4 were co-expressed from one plasmid, respectively. (**C**) Level of H3K27me3 at *Hoxb*, *Eomes, Actb*, and *Ereg* (PP1 to PP3) as determined by ChIP-qPCR in mNSCs. (**D**) Bright-field and GFP fluorescence images of mNSCs 2 days post nucleofection with a dCas9-JMJC_6B-T2A-EGFP-gLacZ plasmid. (**E**) ChIP-qPCR analysis of H3K27me3 around the TSS of *Ereg* and two unrelated genes (*Hoxb5*, *Eomes*) after epigenome editing at the *Ereg* locus. (**F**, **G**) Expression of *Sox2* and *Ereg* as determined by RT-qPCR upon epigenome editing using g*Ereg* EE1 + 2 and g*Ereg* EE3 + 4. Expression normalized to *Gapdh* and relative to g*LacZ* EE. Data information: Scale bar, 100 µm. Bar graphs represent mean values. Error bars represent the SD of three replicates (from two to three independent experiments). One-way ANOVA with Dunnett post hoc test; no statistically significant changes were detected.

 © The Author(s)
